# Pharmacological characterization of mutant huntingtin aggregate-directed PET imaging tracer candidates

**DOI:** 10.1038/s41598-021-97334-z

**Published:** 2021-09-09

**Authors:** Frank Herrmann, Manuela Hessmann, Sabine Schaertl, Karola Berg-Rosseburg, Christopher J Brown, Galina Bursow, Anass Chiki, Andreas Ebneth, Miriam Gehrmann, Nicole Hoeschen, Madlen Hotze, Stefanie Jahn, Peter D Johnson, Vinod Khetarpal, Alex Kiselyov, Karsten Kottig, Stefanie Ladewig, Hilal Lashuel, Sven Letschert, Matthew R Mills, Kathrin Petersen, Michael E Prime, Christoph Scheich, Gerhard Schmiedel, John Wityak, Longbin Liu, Celia Dominguez, Ignacio Muñoz-Sanjuán, Jonathan A Bard

**Affiliations:** 1 Evotec SE, Essener Bogen 7, 22419 Hamburg, Germany; 2 Evotec (U.K.) Ltd., 114 Innovation Drive, Milton Park, Abingdon, OX14 4RZ UK; 3 Laboratory of Molecular and Chemical Biology of Neurodegeneration, Brain Mind Institute, Ecole Polytechnique Fédérale de Lausanne (EPFL), 1015 Lausanne, Switzerland; 4 CHDI Management/CHDI Foundation, 6080 Center Drive, Suite 700, Los Angeles, CA 90045 USA

**Keywords:** Biomarkers, Pharmacology, Diseases of the nervous system

## Abstract

Huntington’s disease (HD) is caused by a CAG trinucleotide repeat expansion in the first exon of the *huntingtin* (*HTT*) gene coding for the huntingtin (HTT) protein. The misfolding and consequential aggregation of CAG-expanded mutant HTT (mHTT) underpin HD pathology. Our interest in the life cycle of HTT led us to consider the development of high-affinity small-molecule binders of HTT oligomerized/amyloid-containing species that could serve as either cellular and in vivo imaging tools or potential therapeutic agents. We recently reported the development of PET tracers CHDI-180 and CHDI-626 as suitable for imaging mHTT aggregates, and here we present an in-depth pharmacological investigation of their binding characteristics. We have implemented an array of in vitro and ex vivo radiometric binding assays using recombinant HTT, brain homogenate-derived HTT aggregates, and brain sections from mouse HD models and humans post-mortem to investigate binding affinities and selectivity against other pathological proteins from indications such as Alzheimer’s disease and spinocerebellar ataxia 1. Radioligand binding assays and autoradiography studies using brain homogenates and tissue sections from HD mouse models showed that CHDI-180 and CHDI-626 specifically bind mHTT aggregates that accumulate with age and disease progression. Finally, we characterized CHDI-180 and CHDI-626 regarding their off-target selectivity and binding affinity to beta amyloid plaques in brain sections and homogenates from Alzheimer’s disease patients.

## Introduction

Huntington’s disease (HD) is an autosomal dominant, progressive neurodegenerative disease that is characterized clinically by cognitive, behavioral, and motor deficits, with broad neuronal loss in the basal ganglia and several cortical areas^1–3^. HD is caused by a CAG trinucleotide repeat expansion in the first exon of the huntingtin (*HTT*) gene coding for the huntingtin (HTT) protein, a 3144 amino acid protein of pleiotropic functions. Expanded HTT has a high propensity to misfold and self-assemble into amyloidogenic species, oligomers and fibrils of different sizes and morphologies. HTT fragmentation that leads to generation of N-terminal fragments bearing the expanded polyglutamine (polyQ) domain is thought to accelerate the formation of higher-order oligomers, fibrils and large inclusion bodies within the nucleus and cytoplasm of brain cells^4^. The different intermediates along the pathway of HTT inclusion formation are thought to be proteotoxic, as reflected by the ensuing cellular dysfunction and degeneration that is accompanied by the expression and aggregation of mHTT in different cellular and animal models of HD^5–11^.

The misfolding and consequential aggregation of mHTT that underpins HD pathology is part of a broader class of proteopathies that include neurodegenerative diseases such as Alzheimer’s disease (AD), Parkinson's disease (PD), amyotrophic lateral sclerosis (ALS), frontotemporal dementia (FTD) and spinocerebellar ataxia 1^12–18^. Currently, there are no known effective treatments for HD that correct protein misfolding. Potential therapeutic strategies include development of agents that can prevent or modify protein misfolding or restore misfolded proteins to their native states and potentially their normal functions. Consequently, an active area of HD research is the search for small molecules that can bind to HTT and prevent misfolding and/or inhibit the formation of toxic oligomeric and high molecular weight mHTT aggregates^19–28^.

Currently, several therapies that aim to lower HTT are in late-stage preclinical and clinical development [reviewed in^29–31^], and antisense oligonucleotides (ASOs) targeting HTT have been shown to lower HTT expression in the CSF of patients in a Phase1/2a study^31–33^. However, there is presently no information concerning the regional effects of HTT-lowering therapeutics in the human brain due to the lack of HTT-specific imaging tools that can assess the impact of regionally restricted therapies. We therefore set out to identify small-molecule binders of mHTT aggregates that could be optimized as an in vivo positron emission tomography (PET) imaging biomarker to provide insight into the progression of HD neuropathology and serve as a potential biomarker for HTT-lowering therapeutic clinical trials, allowing sensitive, quantitative imaging of mHTT non-invasively^29–32^.

Brain imaging may help to bridge the gap in understanding the genetic, cellular, and molecular biological underpinnings of neuropathological dysfunction in Huntington’s disease. Additionally, high affinity mHTT small- molecule binders could be used as warheads for protein degraders (e.g. PROTACs, AUTACs and LYTACs) to promote ubiquitination-dependent, proteasomal-mediated or lysosomal-mediated modulation of HTT levels as a therapeutic strategy.

Various PET radioligands have been developed to quantify amyloid and tau pathology in vivo^34–36^. PET can quantify and localize molecular processes in vivo; amyloid imaging has already facilitated clinical trial design and identified new drug targets^37^. Noteworthy is the absence of PET radioligands specific for other misfolded proteins responsible for a spectrum of age-related neurodegenerative diseases, such as huntingtin, α-synuclein (associated with Parkinson’s disease), and TDP-43 (associated with frontotemporal dementia, amyotrophic lateral sclerosis and several types of encephalopathies). Development of PET tracers specific for these other neuropathologically-associated misfolded proteins would be instrumental in advancing biomarkers that may allow more refined tools to assist in diagnostics, tracking disease progression and monitoring target engagement and disease-modifying therapeutics seeking to alter the levels or states of these proteins.

We previously reported the development of two high-affinity, potent, cell-permeable and selective ligands specific for mHTT aggregates (CHDI-180 and CHDI-626) that could serve as PET imaging tracers^38,39^ using a large set of binding assays we developed and optimized. We used recombinant proteins and mHTT aggregate-containing brain tissue lysates to identify and optimize the properties of small molecules that selectively bind mHTT, without binding activity for unexpanded HTT, versus other amyloid-forming proteins associated with many neurodegenerative brain diseases. mHTT aggregate binders displaying nanomolar affinity were radiolabeled with tritium and used in autoradiography (ARG) studies using brain sections from mouse HD models and human HD post-mortem samples.

Here, we present an in-depth pharmacological characterization of our advanced mHTT aggregate-directed PET imaging tracer candidates CHDI-180 and CHDI-626^38,39^, demonstrating that our methods can be utilized for discovery and development of imaging ligands that may enable a better translation from studies conducted with recombinant proteins and cellular systems to live imaging studies as a strategy to develop in vivo imaging tools for human studies.

## Results

We have recently reported structure–activity studies that led to the discovery of CHDI-180 and CHDI-626 as a potential PET tracer for imaging of aggregated mHTT species^38,39^. Extensive in vitro characterization of CHDI-180 and CHDI-626 showed that the compounds have properties suitable for reaching the CNS with minimal non-specific binding as well as acceptable plasma, microsomal, and hepatocyte stability. First proof-of-concept target engagement studies in brain slices from an HD mouse model and post-mortem human HD samples and in vivo PET studies in an HD mouse model were also reported^38,39^.

**Figure 1:**
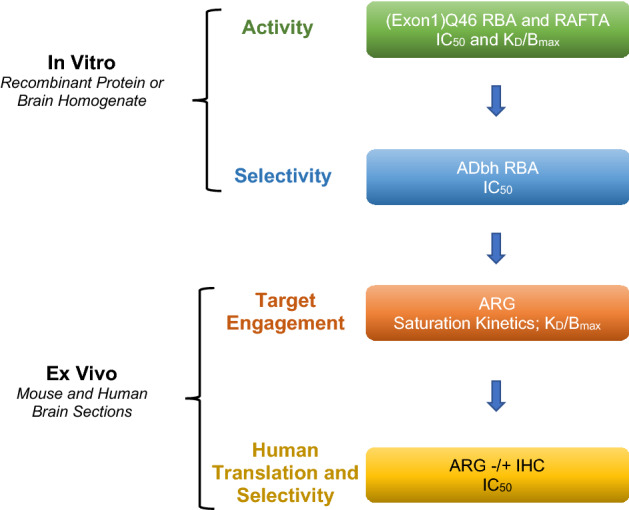
Screening Cascade of in vitro*/*ex vivo pharmacological assays.

For identification of candidates and their comprehensive characterization regarding binding potency, target engagement and selectivity, we have developed various in vitro and ex vivo radiometric binding assays using recombinant mHTT aggregates, mHTT aggregate-containing brain lysates and brain sections of mouse HD models and human post-mortem samples. The progression of compound selection and characterization followed the screening cascade shown in Fig. 1.

Briefly, compounds were first tested for their binding affinity to the (Exon1-)Q46-aggregates in a competition Radioligand Binding Assay (RBA). Potent binders were further triaged based on their selectivity against beta amyloid/tau aggregates in post-mortem AD patient brain homogenates (ADbh RBA). Promising compounds were then labeled with tritium isotope and evaluated for target binding in brain sections from mouse HD models and human post-mortem samples by autoradiography (ARG).

Additional assays were developed, e.g. Radiometric Filter Trap Assay (RAFTA) and High Resolution Autoradiography (HR ARG), to enable in vitro/ex vivo translation and further characterization of the selected radioligands, and facilitating the investigation of whether there was a correlation between radioligand binding (assessed by ARG and RAFTA) and mHTT aggregate load (determined by IHC and an aggregate-directed quantitative MSD assay).

### Characterization of small molecule interactions to recombinantly-derived HTT aggregates by direct and competition binding

We reasoned that the readily aggregating mHTT Exon-1 fragment (polyQ expansion > 39) could serve as a proxy for the pathological mHTT and facilitate screening to identify high-affinity HTT small-molecule binders. In the RBA, test compounds were incubated with recombinant polyQ-aggregates in the presence of a suitable radioligand, and the concentration response of inhibition of the radioligand binding to such aggregates was used to derive the competition binding potency (IC_50_), which was used for structure–activity relationship (SAR) analysis and compound selection.

Two different forms of recombinant polyQ peptides, Q46 and HTT Exon1-Q46, were used as surrogates for the endogenous mHTT target in the RBA. These two proteins form aggregates large enough to be trapped on a glass-fiber filter plate, once the N-terminal tags (GST and MBP, respectively) are cleaved off by thrombin digestion (see Supplementary Fig. S2A). In the assay, bound radioligand can be separated from unbound by filtration and washing, as applied in the RBA and radiometric filter trap assay (RAFTA; see Supplementary Fig. S4A).

In order to characterize the aggregates (fibrils, as defined by Thioflavin S (ThS) staining; see Supplementary Fig. S2), we analyzed the uncleaved soluble proteins (Supplementary Fig.S2B, fraction 1), the cleaved proteins after thrombin addition and initiation of aggregation in situ (fraction 2), as well as the pellet (fraction 3) after centrifugation to separate the fibrils from the fusion protein and any remaining solution protein (fraction 4). As expected, GST-Q46 and MBP-Exon1-Q46 did not show any significant aggregate/fibril formation before the cleavage of the fusion protein (GST or MBP), as indicated by the low ThS fluorescence signal (Supplementary Fig. S2C), by the absence of any retained aggregates in the filter-trap assay (Supplementary Fig. S2D), and by electron microscopy analysis (Supplementary Fig. S2E). After cleavage of the tags from the fusion proteins, both Q46 and Exon1-Q46 proteins rapidly formed fibrils that were ThS-positive (Supplementary Fig. S2C) in both the crude aggregates and the pellet after centrifugation. The filter-trap assay and electron microscopy confirmed the presence of the fibrils in the crude aggregate and the pelleted fibrils (Supplementary Fig. S2E). Interestingly, the fibrils formed from Q46 showed clumped aggregates as previously described^40^, unlike Exon1-Q46, which formed single and separated fibrils^41^.

To identify novel HTT aggregate binders, an initial screen of ~ 1600 compounds, consisting of a focused set with similarity to known beta amyloid and beta-sheet binders^39^, was conducted with the RBA using Q46 aggregates and an analog of the known beta amyloid binder FDDNP (applied at 140 nM) (**8**) as the radioligand^42^. The radioligand bound to the aggregates with a K_d_ of 139.5 nM (Table 1). For screening, the compounds were tested in competition binding experiments at two concentrations (1 µM and 0.1 µM) and compounds displaying > 80% displacement of **8** at 1.0 µM were considered as hits. A total of 160 small molecule scaffolds (14 at 0.1 µM) were identified in the hit identification campaign corresponding to a hit rate of 9.9% (0.9%).

**Table 1:** Binding and competition potencies in RBA.

Compound ID	K_d_ (nM)		IC_50_ (nM)	
	Exon1-Q46	Q46	Exon1-Q46	Q46
**1** (CHDI-180)	2.6 ± 0.9	7.7 ± 3.3	1.3 ± 0.7	1.2 ± 0.5
**2** (CHDI-626)	4.5 ± 0.8	5.8 ± 1.7	2.9 ± 0.6	3.6 ± 1.6
**3**	2.5 ± 0.6	4.7 ± 0.2	1.2 ± 0.4	1.8 ± 0.8
**4**	5.5 ± 1.3	7.8 ± 4.1	0.4 ± 0.1	0.7 ± 0.1
**5**	5.2 ± 1.8	8.7 ± 4.0	5.8 ± 1.7	4.7 ± 2.4
**6** (PiB)	(–)^a^	(–)^a^	72.5 ± 0.4	> 333
**7** (T808)	(–)^a^	(–)^a^	> 333	> 333
**8**	n.a	139.5 ± 36.9*	9.9 ± 1.0	10.3 ± 2.7

Mean values and standard deviations; see structures of compounds in Supplementary Fig. S1. *Higher protein concentrations (10–30 µM) were applied. ^a^Not determinable because TB ≅ NSB.

From that effort, compounds displaying low nM binding affinity to mHTT aggregates were identified and selected as next generation radioligands^39^. Both binding molecules and assay conditions were further optimized resulting in the RBA assay described here using 0.3 nM of tritiated compound **3** as radioligand and much lower protein aggregate concentrations (1 µM) compared to the RBA conditions used for screening (33 µM).

**Figure 2:**
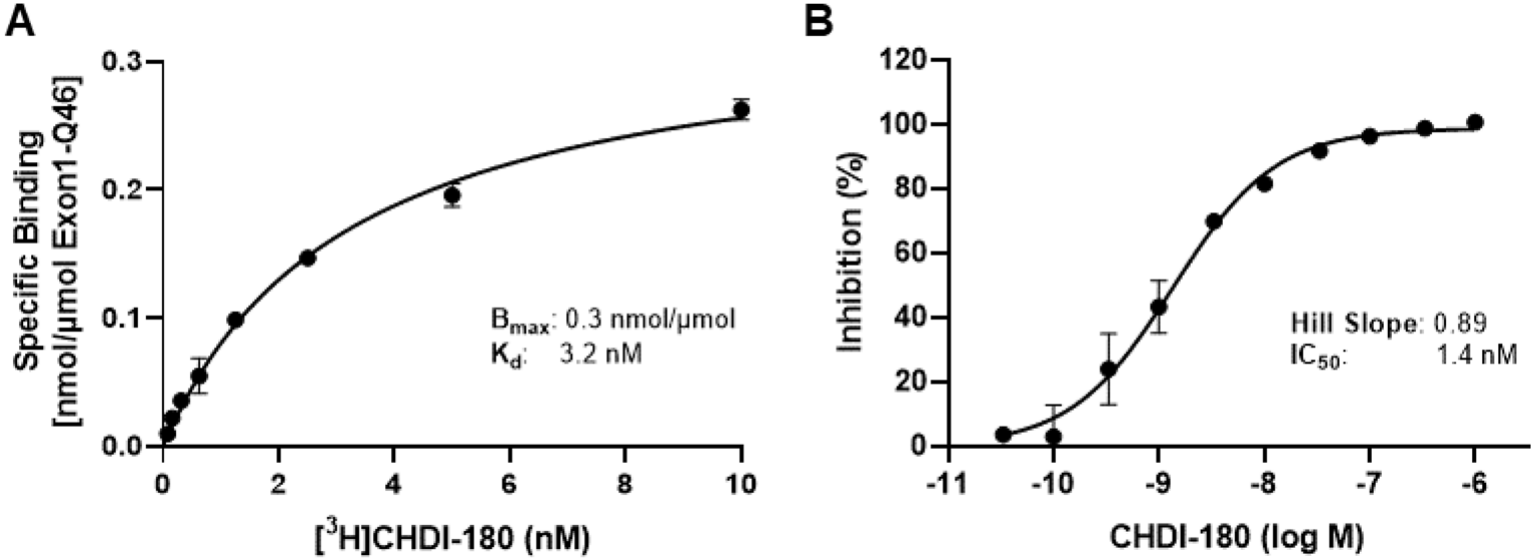
RBA applications. (**A**) Saturation binding to determine maximal binding (B_max_) and binding affinity (K_d_) values of radioligand CHDI-180 for binding to recombinant Exon1-Q46 aggregates. (**B**) Competition binding of CHDI-180 against tritiated **3** to determine IC_50_ value of compound. Note: Representative graphs of single experiments with triplicate data points are shown. Mean K_d_ and IC_50_ values (n = 5) for CHDI-180 are shown in Table 1.

The RBA was applied to determine radioligand displacement potencies (IC_50_ values) of unlabeled small molecules, using tritiated tool compound **3** as a radioligand and to measure binding affinity of radiolabeled compounds (K_d_ values), such as [^3^H]CHDI-180 (Fig. 2; Table 1). The assay had a robust performance with Z’ > 0.5 and a signal window (TB/NSB) > 3 and served as a first-tier assay to establish a structure–activity relationship of mHTT aggregate-binding compounds. At this first stage, compounds were mainly selected based on their binding affinity to the Exon1-Q46-aggregates. Saturation binding kinetics for [^3^H]CHDI-180 in the RBA revealed average K_d_ values for Exon1-Q46- and Q46-derived aggregates of 2.6 nM and 7.7 nM, respectively. A summary of binding and competition potencies of compounds discussed in this article is shown in Table 1.

### Pharmacological characterization of [^3^H]CHDI-180 binding to HTT aggregates in HD mouse models

One of the unknowns in conducting binding assays with either mHTT recombinantly-derived proteins or HD brain-derived homogenates is whether the binding epitopes and/or protein conformations are retained during the generation and extraction protocols and represent those that are physiologically expressed in HD.

**Figure 3:**
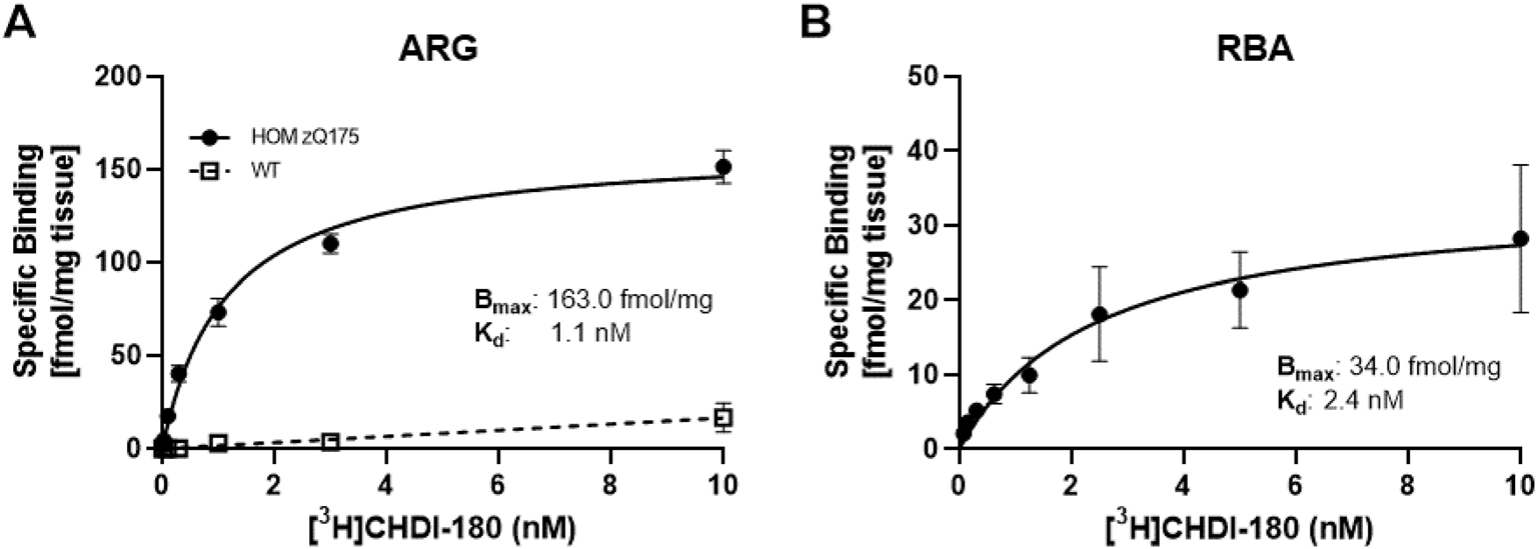
Saturation binding of [^3^H]CHDI-180 using HOM zQ175 (12 months) HD mouse brain. (**A**) in vitro autoradiography (ARG) in cortex. (**B**) Whole brain homogenate RBA. Specific binding normalized per mg tissue. Representative single experiments with replicate data points.

To address this, we measured in situ binding of [^3^H]CHDI-180 by autoradiography (ARG) to determine if the binding pharmacology we observed with RBA aligns to the profile obtained with mHTT aggregates in situ and expressed in HD pathology. Autoradiographic saturation binding studies were performed in coronal half-brain sections from 12-month-old homozygous (HOM) zQ175 HD^43,44^ and WT mice. For ARG, we chose 12-month-old HOM Q175 as they have one of the highest expressed aggregate load of HD animal models (unpublished data). Binding of [^3^H]CHDI-180 was quantified by densitometric analysis and specific binding was determined (Fig. 3A). The affinity (K_d_) of [^3^H]CHDI-180 was similar in ARG experiments (K_d_ = 1.1 nM) with zQ175 HD mouse brain slices compared to RBA measurements using recombinant Exon1-Q46 (K_d_ = 2.6 nM; see Table 1) or zQ175 brain homogenates (K_d_ = 2.4 nM; Fig. 3B). However, with the RBA, only a low binding signal B_max_ (Fig. 3B) was measured with homogenates from HOM zQ175 brains when compared with B_max_ observed in ARG (Fig. 3A) and no appropriate binding signal could be generated with the RBA using R6/2 brain homogenates (data not shown). This suggests that only a minor fraction of the endogenous mutant HTT target species detected by ARG is retained on the filter plate in the RBA assay and smaller aggregate species are potentially washed through the pores and lost in the RBA assay.

To allow satisfactory signal-to-background detection using brain homogenate-derived aggregates and to circumvent the possible flow-through of smaller aggregates using the RBA filter plate, we established a novel Radiometric Filter Trap Assay (RAFTA), which is based on a classical filter trap or retardation protocol^45,46^ and uses a conventional filter membrane and subsequent exposure to phosphor imaging for readout instead of using the plate/liquid scintillate paradigm that was used for RBA. In this assay, aggregate-containing brain homogenates are incubated with the radioligand and vacuum filtered through GF/F membrane filters with smaller pore sizes (0.7 µm) than those from the aforementioned and conventional RBA filter plate (i.e. pore size of 1–1.2 µm) enabling higher assay sensitivity by retention of smaller mHTT species present in complex matrices, such as lysates from cellular and animal HD models. First, we validated the assay by testing binding affinity of [^3^H]CHDI-180 to recombinant Exon1-Q46 aggregates. In RAFTA saturation binding experiments, we observed a K_d_ = 5.6 nM (Supplementary Fig. S4A) which correlated well with data obtained from RBA experiments using recombinant Exon1-Q46 aggregates (K_d_ = 2.6 nM; see Table 1).

Due to significant increase in signal we observed with the RAFTA using Exon1-Q46 aggregates (e.g., B_max_ = 1456 fmol/mg; Supplementary Fig. S4A), the utility of the RAFTA format was extended to explore the ability to detect aggregates in the HET zQ175 model, which expresses significantly less aggregates than its age-matched homozygous counterpart due to gene dose of the mHTT allele^43,44^. We fractionated 3 different brain subregions (e.g., cortex (CTX), striatum (STR) and hippocampus (HPC)) into pellet or supernatant material after centrifugation of brain homogenates. As shown in Supplementary Fig. S4B, pelleted materials of cortices, striata or hippocampi from 9- month-old heterozygous (HET) zQ175 mice showed greater signal (> tenfold) after binding of 3 nM [^3^H]CHDI-180 compared to analogous material from age-matched WT mice. We also interrogated the supernatants to evaluate whether there may be any forms of mHTT that did not pellet using our protocol but that could still be retained on the membrane and able to be bound by the radiolabeled small molecule. Although there was some signal, indicative of binding of [^3^H]CHDI-180 to supernatants from all 3 brain subregions of HET zQ175 mice, this binding was lower than that observed for the pellet fraction in each subregion of HET zQ175 mice (Supplementary Fig. S4B).

These data are consistent with [^3^H]CHDI-180 binding to both the recombinant HTT aggregates and mHTT aggregates formed in the brains of zQ175 HD animals and support the use of the RAFTA assay to help characterize and quantify the binding of small molecules to brain homogenates from HD animal models.

### Age-dependent increase of [^3^H]CHDI-180 binding in HOM zQ175 HD mouse brains

**Figure 4:**
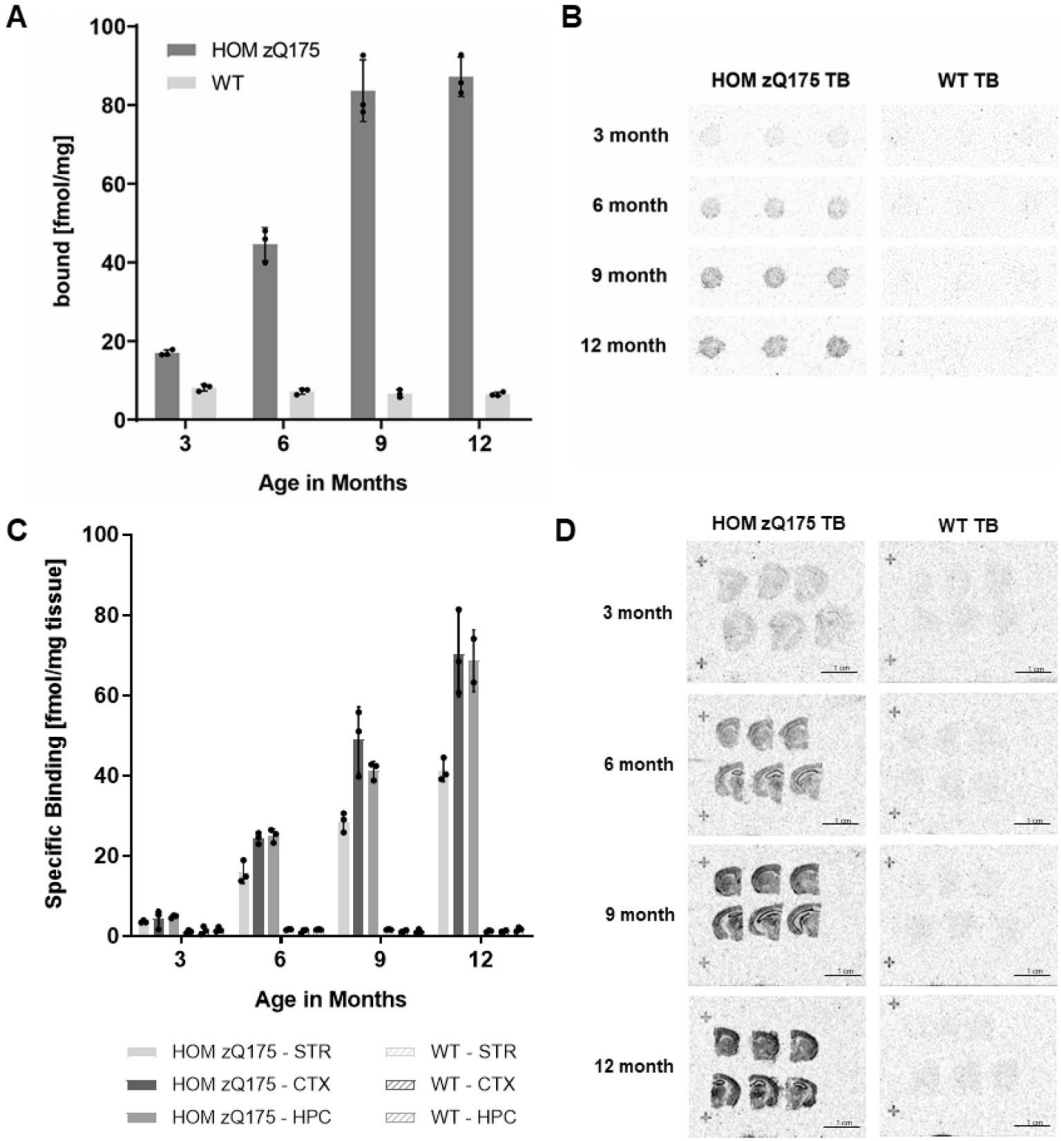
Age-dependent increase of [^3^H]CHDI-180 binding in HOM zQ175 HD mouse brains. (**A**) Radiometric filter trap assay (RAFTA) showing an age-dependent increase in [^3^H]CHDI-180 binding to aggregates in brain homogenates of HOM zQ175 HD mice. (**B**) Example images of [^3^H]CHDI-180 binding in RAFTA. (**C**) In vitro ARG showing an age-dependent increase in [^3^H]CHDI-180 binding to brain sections of HOM zQ175 HD mice. Specific binding (SB) of 0.5 nM [^3^H]CHDI-180 in striatum (STR), cortex (CTX) and hippocampus (HPC) of 3-, 6-, 9- and 12-month-old HOM Q175 mice and age-matched WT mice. (**D**) Example images of [^3^H]CHDI-180 binding. Data are depicted as mean ± SD from a total of n = 3 brain hemispheres per age and genotype.

Aggregates of mHTT accumulate in HD mouse models such as R6/2 and zQ175 (i.e. increase in quantity and size) in an age-, disease-, and region- progressive fashion^47–50^ and hence the specific binding signal of a small molecule targeting mHTT aggregates is expected to increase with age in brains of these animals. Therefore, homogenates or slices from brains of 3-, 6-, 9- and 12-month-old HOM zQ175 and age-matched WT mice were prepared and subjected to RAFTA or ARG analysis using [^3^H]CHDI-180; HOM zQ175 mice were used due to increased sensitivity and dynamic range compared to HET, likely reflecting the greater target load of the former versus the latter model. As shown in Fig. 4A and B, RAFTA experiments showed an age-dependent increase in [^3^H]CHDI-180 binding in brain homogenates of HOM zQ175 HD mice. Little-to-no background signal was detectable in brain homogenates from WT mice of all ages.

In ARG experiments, performed on coronal brain sections from 3-, 6-, 9- and 12-month old HOM zQ175 HD and WT mice, specific binding (SB) of [^3^H]CHDI-180 was quantified by densitometric analysis in three brain regions (CTX, STR, and HPC). Figure 4C and D display autoradiography data demonstrating an age-and region-dependent increase of specific [^3^H]CHDI-180 binding in STR, CTX and HPC in brain sections from HOM zQ175 HD mice, corroborating the RAFTA data (Fig. 4A and B). Although a SB signal, as measured by displacement assays with cold compound, was detectable in brain sections from WT mice, this signal was at very low intensity levels compared to HD mice and uniformly distributed within the tissue. The quantification of total binding (TB), non-specific binding (NSB) and SB for each age group and genotype for each of the three brain subregions is summarized in Supplementary Table S2.

Neither TB nor NSB signals increased in brain sections from WT mice in an age-progressive fashion and remained unchanged over the evaluated ages, further supporting mHTT aggregates as target of [^3^H]CHDI-180 binding. The ARG data are in accordance with in vitro binding using recombinant Exon1-Q46 aggregates in the RBA format (Table 1) and binding to mHTT aggregates in brain homogenates from HD mouse models using the RAFTA assay (see Fig. 4A and Supplementary Fig. S4).

These results are consistent with the hypothesis that [^3^H]CHDI-180 specifically bound mHTT, consistent with aggregates (see next section) expressed in STR, CTX and HPC from HD mice brain sections that accumulate (i.e., increase in quantity and size) in an age- and disease-progressive fashion^47,49,50^.

### Correlation of CHDI-180 binding with mHTT aggregate load in HD mouse brains

We were interested in exploring whether the age- and disease-progressive binding of CHDI-180 to mouse HD brains shown in Fig. 4 was likely directed to an aggregated state of mHTT and not to monomeric or non-aggregate mHTT.

To accomplish this, we included immunohistochemistry (IHC), utilizing an antibody directed to mHTT aggregates (mEM48)^51^, and the previously established quantitative sandwich-based immunoassay-type HTT assays (Meso Scale Discovery, MSD), directed to either soluble, expanded mHTT^48^ or aggregated mHTT^44^. Our approach was to investigate if there was a correlation between radioligand binding assessed by ARG and RAFTA using [^3^H]CHDI-180, and mHTT aggregate load determined by IHC and an aggregate-directed quantitative MSD assay. Additionally, we analyzed samples using the quantitative soluble expanded mHTT MSD assay to better understand the relationship between small molecule binding and different states of mHTT (Supplementary Fig. S5B).

**Figure 5:**
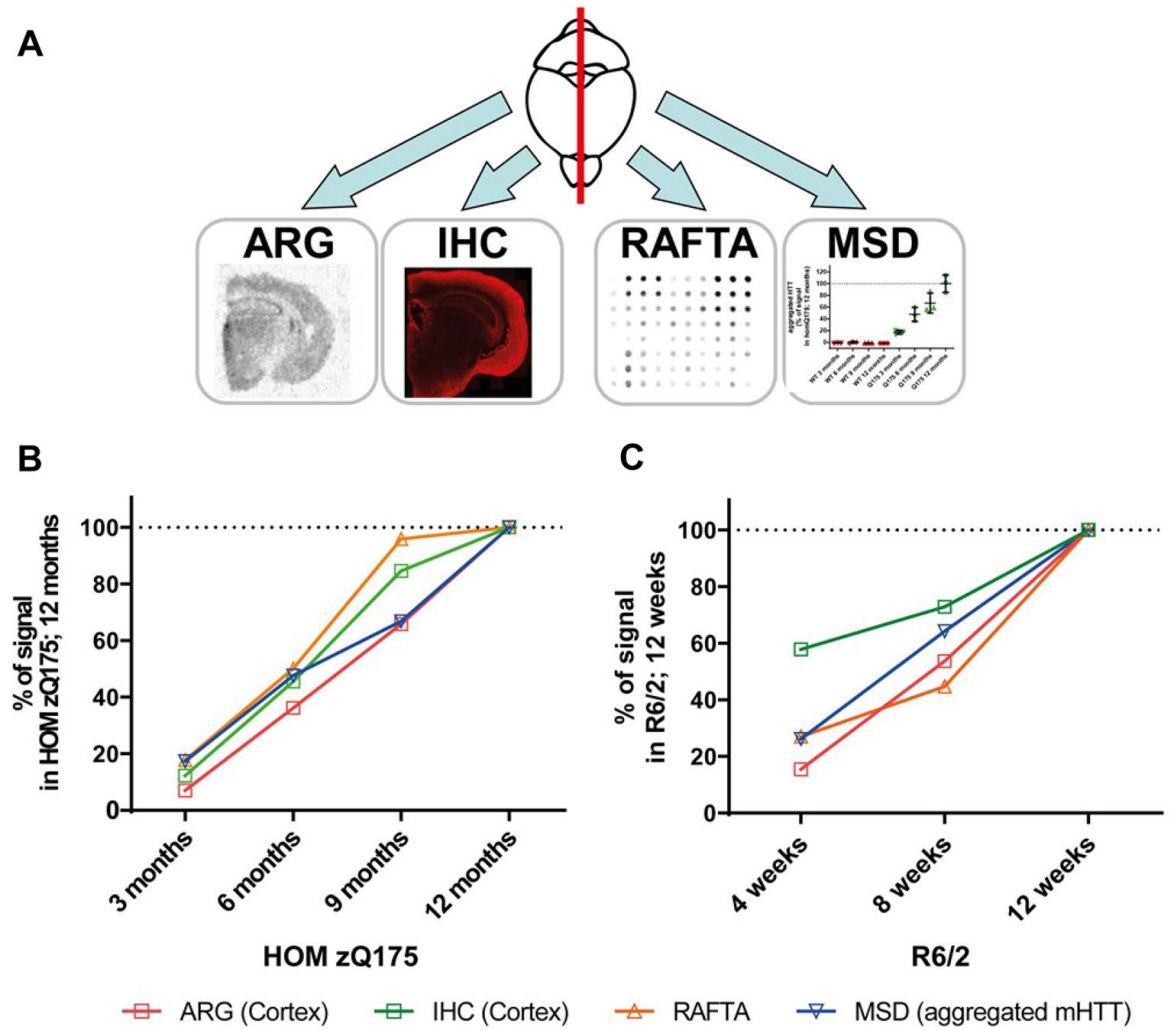
Correlation of CHDI-180 binding and aggregate load by ARG, IHC, RAFTA and MSD quantitation. (**A**) Schematic experimental overview. Brains from 3, 6, 9 and 12-month-old HOM zQ175 or 4, 8 and 12-week-old R6/2 and age-matched WT mice (n = 3) were split into halves to separate the hemispheres. One hemisphere per brain was assigned to sectioning for analysis by autoradiography and IHC, the second hemisphere was homogenized for analysis by RAFTA and MSD assays. Correlation of [^3^H]CHDI-180 binding in ARG and RAFTA and aggregate load determined by IHC and MSD quantitation^44^ in (**B**) HOM zQ175 and (**C**) R6/2 mice. Data is normalized to % of the signal in 12-month-old HOM zQ175 or 12-week-old R6/2 animals, respectively. Note that for autoradiography and IHC data only CTX tissue was used.

Brains from 3, 6, 9 and 12-month-old HOM zQ175 and age-matched WT mice were split into halves to separate the hemispheres. One hemisphere per brain was assigned to sectioning for analysis by ARG and IHC, the second hemisphere was homogenized for analysis by RAFTA and MSD assays (Fig. 5A).

For IHC, adjacent coronal brain sections to the ones used in ARG experiments described above (see Fig. 4C) were immunohistochemically stained with mEM48 mHTT aggregate-directed monoclonal antibody to quantify the mHTT aggregate load in the different regions of interest at different ages (Supplementary Fig. S5A). The second separated hemisphere of each individual brain was homogenized and subjected to RAFTA analysis (Fig. 4A, B) and two quantitative MSD immunoassays (Supplementary Fig. S5B), one to assess the levels of soluble, expanded mHTT, and the other to measure the levels of aggregated mHTT^44,48^.

As stated above, using ARG (Fig. 4C), [^3^H]CHDI-180 showed an age-dependent increase in specific binding to STR, CTX and HPC from HOM zQ175 mice brains which was also confirmed by RAFTA analysis of corresponding brain hemispheres (Fig. 4A, B). IHC analysis of adjacent brain sections with the mHTT aggregate-directed monoclonal antibody mEM48 (Supplementary Fig. S5A) and determination of aggregated HTT levels in brain homogenates by the aggregate-specific MSD assay (MW8/4C9-ST; Supplementary Fig. S5B, right) similarly revealed the age-dependent increase of HTT aggregates in the HOM zQ175 HD mouse model (Fig. 5B). Note that the soluble, expanded mHTT MSD assay (Supplementary Fig. S5B, left) showed an age-dependent decrease of mHTT in the same material, as previously reported for HET zQ175 mice^48^.

These data strongly indicate that [^3^H]CHDI-180 binds specifically to mHTT aggregates in the brain from HOM zQ175 mice; that is, the binding correlated better with an increase in age-dependent mHTT aggregate load rather than with the decrease in age-dependent soluble mHTT, observed in 3-, 6-, 9- and 12-month old HOM zQ175 mice. The lower binding of [^3^H]CHDI-180 to WT compared to HD brains in RAFTA and ARG experiments (see Fig. 4) also suggests that this small molecule has minimal binding to WT (unexpanded) HTT protein and is consistent with the use of fibrils (expanded mHTT) as the screening target for its discovery.

To corroborate the results with an alternative HD mouse model, analogous experiments were performed using brains from 4-, 8- and 12-week-old R6/2 mice. In this transgenic HD mouse model, [^3^H]CHDI-180 also showed an age-dependent increase in specific binding to STR, CTX and HPC brain sections using ARG and in corresponding brain hemispheres by RAFTA and aggregate-specific MSD analysis (Fig. 5C and Supplementary Fig. S9A).

### Binding of radiolabeled CHDI-180 in brain sections from human post-mortem HD brain

Next, we extended the observed in situ binding from mouse HD model-derived tissues to humans to provide information about the general translatability of this mHTT aggregate binder, and specifically with regards to pathology-specificity and species-selectivity.

**Figure 6:**
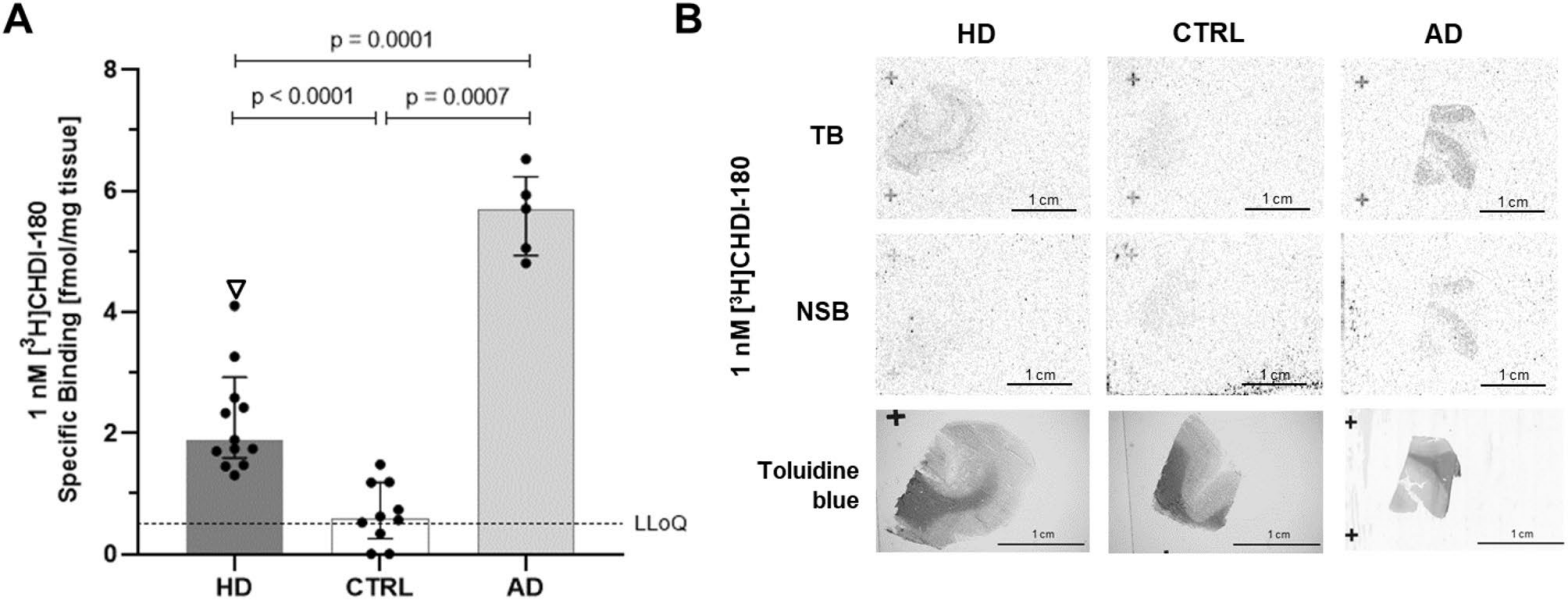
ARG studies investigating CHDI-180 ligand binding to human HD, AD and CTRL brain sections. (**A**) Specific binding in gray matter of [^3^H]CHDI-180 to human brain sections (frontal cortices) in in situ ARG. Black circles indicate binding to individual adult HD brains, the white triangle depicts binding to one juvenile HD case. Data shown as median ± interquartile range. Statistical analysis by Mann–Whitney test, two-tailed, 95% confidence level. LLoQ: lower limit of quantification (**B**) Representative images of [^3^H]CHDI-180 total binding (TB) and non-specific binding (NSB) in human HD, AD and CTRL brain sections. Toluidine blue staining was used to discriminate white matter (dark gray) and gray matter (light gray) in the same section used for autoradiography.

We have previously presented data of [^3^H]CHDI-180 showing mHTT-specific binding in post-mortem human HD brains^39^. We extended this observation using the in situ binding assay (ARG) and additional human post-mortem frontal cortex brain tissues from HD, AD and CTRL subjects (see Fig. 6). Because of progressive neurodegeneration in HD striatum, we decided to use frontal cortex tissue in our binding studies since that region also expresses mHTT aggregates but is not as much impacted by neuronal loss. Examples of autoradiograms of TB and NSB of 1 nM [^3^H]CHDI-180 in frontal cortex brain sections from selected adult HD, CTRL and AD donors are shown in Fig. 6B; quantification data are depicted as specific binding in gray matter in Fig. 6A (n = 12, n = 10 and n = 5, respectively; for demographic information, see Supplementary Table S1). We wanted to investigate if [^3^H]CHDI-180 shows different binding properties in different forms of the disease. To address this, we included juvenile HD (one case) in addition to post-mortem adult-onset HD brains; juvenile HD is characterized by an early onset and much faster progression compared to adult HD.

Confirming previous data reported by our group, [^3^H]CHDI-180 showed a low but significantly greater binding to frontal cortical sections from HD donors compared to CTRL tissue (2.0 ± 0.4 fmol/mg tissue for HD vs. 0.8 ± 0.3 for CTRL; p < 0.01, comparing HD vs. CTRL)(Fig. 6A). [^3^H]CHDI-180 showed distinct specific binding in gray matter areas with very low white matter binding (≤ 0.5 fmol/mg tissue). These data are in accordance with the observation that mHTT aggregates are primarily found in cortical neurons (gray matter) and infrequently expressed in white matter^52^. Even though [^3^H]CHDI-180 showed the highest binding density to the juvenile HD brain, similar [^3^H]CHDI-180 binding densities were observed between adult and juvenile onset HD cases, indicating that the compound recognizes mHTT aggregates equally in both forms of the disease.

Importantly, since we derived our mHTT-aggregate binder [^3^H]CHDI-180 from known PET tracers targeting beta amyloid plaques, which are known to have β-sheet structures that are common features of aggregated proteins, it is not surprising that binding of [^3^H]CHDI-180 in AD post-mortem brain tissues was significantly greater than in HD (5.6 ± 0.7 fmol/mg tissue)(Fig. 6A). The binding level is extremely low compared to known beta amyloid binders (e.g. PiB, SB ~ 2,800 fmol/mg tissue; Supplementary Fig. S6A), but significantly greater than to CTRL sections (p < 0.01). Noteworthy is that NSB of [^3^H]CHDI-180 was below 0.5 fmol/mg tissue in HD and CTRL brain sections suggesting significant displaceable binding of [^3^H]CHDI-180 with cold compound and therefore TB represented mostly SB.

Although the average age of motor onset for HD (30–50 years) is much younger than that of cognitive decline for the AD population (> 65 years) and the beta amyloid burden in HD patients would be expected to be minimal for detection by [^3^H]CHDI-180, we set out to further improve off-target selectivity and reduce binding affinity to pathological proteins (beta amyloid plaques and/or tau tangles) from the brain homogenate of Alzheimer’s disease patients; this enhanced selectivity would allow potential utility in older HD cohorts that may co-express additional pathologies or co-morbidities.

### Identification of mHTT aggregate binders with reduced binding potency to beta amyloid

To assess aggregate selectivity, we adapted the HTT RBA assay to assess binding of small molecules to aggregate-containing material from post-mortem human AD brain homogenates (e.g., beta amyloid plaques and neurofibrillary tangles) (Supplementary Fig. S7).

**Figure 7:**
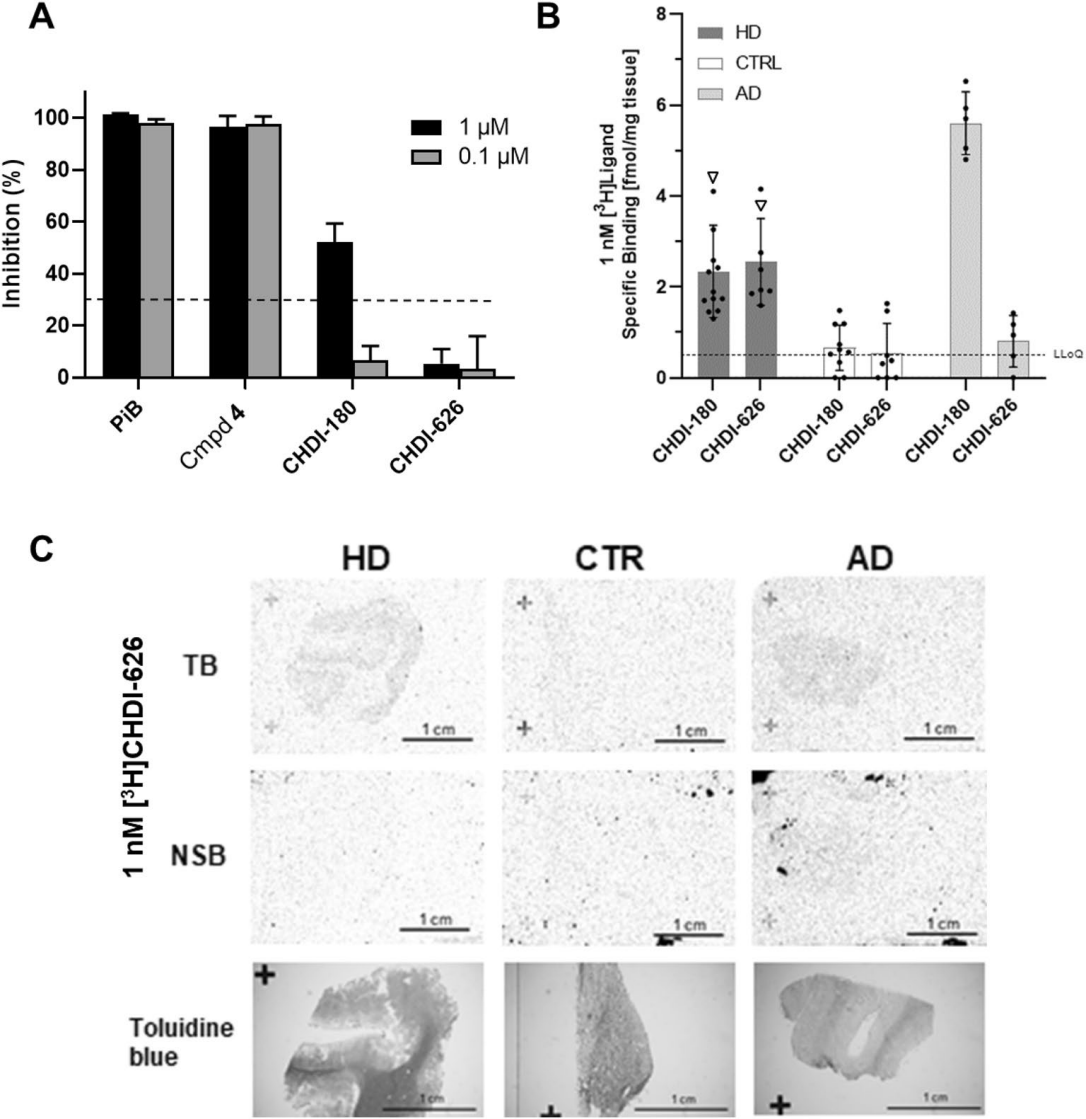
Reduced binding of CHDI-626 to human AD brain. (**A**) ADbh RBA. Competition format against **4** to determine inhibition of compounds at 1 µM and 0.1 µM, applied as counter assay; the dotted line at 30% inhibition indicates the threshold above which a compound is flagged as a potential beta amyloid binder. (**B**) Comparative ARG studies investigating ligand binding to human HD, AD and CTRL brain sections. Total (TB) and non-specific (NSB) of [^3^H]CHDI-180 and [^3^H]CHDI-626 to human brain sections in ARG. Black circles indicate binding to individual adult HD brains, the white triangle depicts binding to one juvenile HD case. Data shown as Median ± interquartile range. Statistical analysis by Mann–Whitney test, two-tailed, 95% confidence level. LLoQ: lower limit of quantification (**C**) Representative images of [^3^H]CHDI-626 total binding (TB) and non-specific binding (NSB) in human HD, AD and CTRL brain sections. Toluidine blue staining was used to discriminate white matter (dark gray) and gray matter (light gray) in the same section used for autoradiography.

Binding of the known beta amyloid ligand [^3^H]PiB (**6**)^53,54^ to human brain homogenate, prepared from AD patients at Braak stages 5–6, was measured in saturation binding experiments to validate the assay (Supplementary Fig. S7A). We observed values of K_d_ (3.4 nM) and B_max_ (10.5 pmol/mg) in our assay, which agreed with data reported in the literature^53,54^. To assess binding to beta amyloid targets, we tested our radiolabelled mHTT ligands for direct binding to AD brain homogenates. Two of the strongest binders were **4** and **5**, and heterologous competition experiments indicated that the tau-selective PET ligand T808 could not compete with **4**, but displaced ~ 50% of bound **5**, indicating that **5** also binds to neurofibrillary tangles whereas **4** is specific for beta amyloid. However, both beta amyloid-specific PET ligands PiB (**6**; Fig. 7A) and Flutemetamol^55^ (data not shown) showed complete displacement of both radioligands. When measuring direct binding to human AD brain homogenate, **4** provided highest sensitivity with a K_d_ of 3.0 nM and B_max_ = 3.7 pmol/mg in the ADbh RBA assay (Supplementary Fig. S7A). In addition, specific binding to human AD brain was confirmed by ARG for **4** (Supplementary Fig. S10) and [^3^H]PiB (Supplementary Fig. S6A).

A competition assay format was applied with **4** as a radioligand (replacing the initially used **5**) to assess competition potency of test compounds that may reflect specificity against beta amyloid/tau aggregates in AD patient brain homogenates (i.e. skewed toward beta amyloid aggregates). As shown in Supplementary Figure S7B, cold **4** was able to displace 0.5 nM **4** with an IC_50_ of 2.5 nM, which is close to its K_d_ value of 3.0 nM (Supplementary Fig. S7A). The competition format was then utilized as a counter screen to interrogate binding to AD pathological aggregates. In this counter screening mode, compounds were applied only at single concentrations of 0.1 µM or 1.0 µM, and compounds displaying less than 30% inhibition at 1.0 µM were considered selective for mHTT aggregate binding. As expected, beta amyloid binder PiB and **4** were able to fully displace **4** even at 0.1 µM (Fig. 7A). The AD liability of [^3^H]CHDI-180 was also observed in the AD RBA by showing an inhibition of ~ 50% at 1 µM, confirming its affinity for pathological aggregates which are present in the AD brains.

In order to understand the SAR around HTT versus beta amyloid/tau aggregate-directed binding and to improve specificity for mHTT, we continued our adaptation of known beta-sheet binders from the Alzheimer’s field and identified a new chemotype with potent binding affinity for recombinant mHTT-derived aggregates, good specific binding in the R6/2 mouse model of HD and brain free fractions predictive of low non-specific binding^38^.

The inclusion of a BIP tricyclic core from T808 (**7**) resulted in the development of the new ligand CHDI-626 (**2;** for structure, see Supplementary Fig. S1) with improved off-target selectivity as shown by the lower binding affinity in the ADbh RBA (Fig. 7A) with a maximum inhibition of < 5% at 1 µM but retaining high affinity for HTT aggregates in RBA and ARG (Table 1; Supplementary Fig. S8). As seen for CHDI-180 (Supplementary Fig. S9A), CHDI-626 showed an age-dependent increase in specific binding to STR, CTX and HPC from R6/2 mice brains using ARG (Supplementary Fig. S9B).

The reduced AD liability of CHDI-626 was further confirmed in human post-mortem brain tissues from HD, AD and CTRL subjects using in situ autoradiography. As shown in Fig. 7B, [^3^H]CHDI-626 exhibited significantly higher binding to sections from HD donors compared to CTRL tissue (2.5 ± 1.0 fmol/mg tissue for HD vs. 0.5 ± 0.7 for CTRL; *p* < 0.01, comparing HD vs. CTRL). [^3^H]CHDI-626 showed one of the highest binding densities to the juvenile HD brain. Similar [^3^H]CHDI-626 binding densities were observed between adult and juvenile onset HD cases. Furthermore, the overall HD brain binding was very similar compared to [^3^H]CHDI-180 indicating that both compounds recognize mHTT aggregates with similar binding properties. However, binding of [^3^H]CHDI-626 in AD post-mortem brain tissues was significantly reduced compared to [^3^H]CHDI-180 (0.8 ± 0.6 fmol/mg for CHDI-626 vs. 5.6 ± 0.7 for CHDI-180), and close to CTRL levels, corroborating the results of the ADbh RBA. Exemplary autoradiograms of TB and NSB of 1 nM [^3^H]CHDI-626 of frontal cortex brain sections from selected HD, CTRL and AD donors are shown in Fig. 7C.

### High resolution microscopic emulsion autoradiography: co-registration analyses in human AD brain sections

To better understand the off-target selectivity and binding nature of CHDI-180 and CHDI-626 in human AD brain tissue, a protocol to investigate co-registration of specific compound binding with either 6E10 antibody, that is reactive to amino acid residues 1–16 of beta amyloid, or the phosphoTau (Ser202, Thr205)-specific antibody AT8, was developed. The technique referred to as high-resolution microscopic emulsion autoradiography (HR ARG) is the combination of radioligand binding and recognition of the target structure at the cellular level by IHC to evaluate co-registration of both detection molecules.

**Figure 8:**
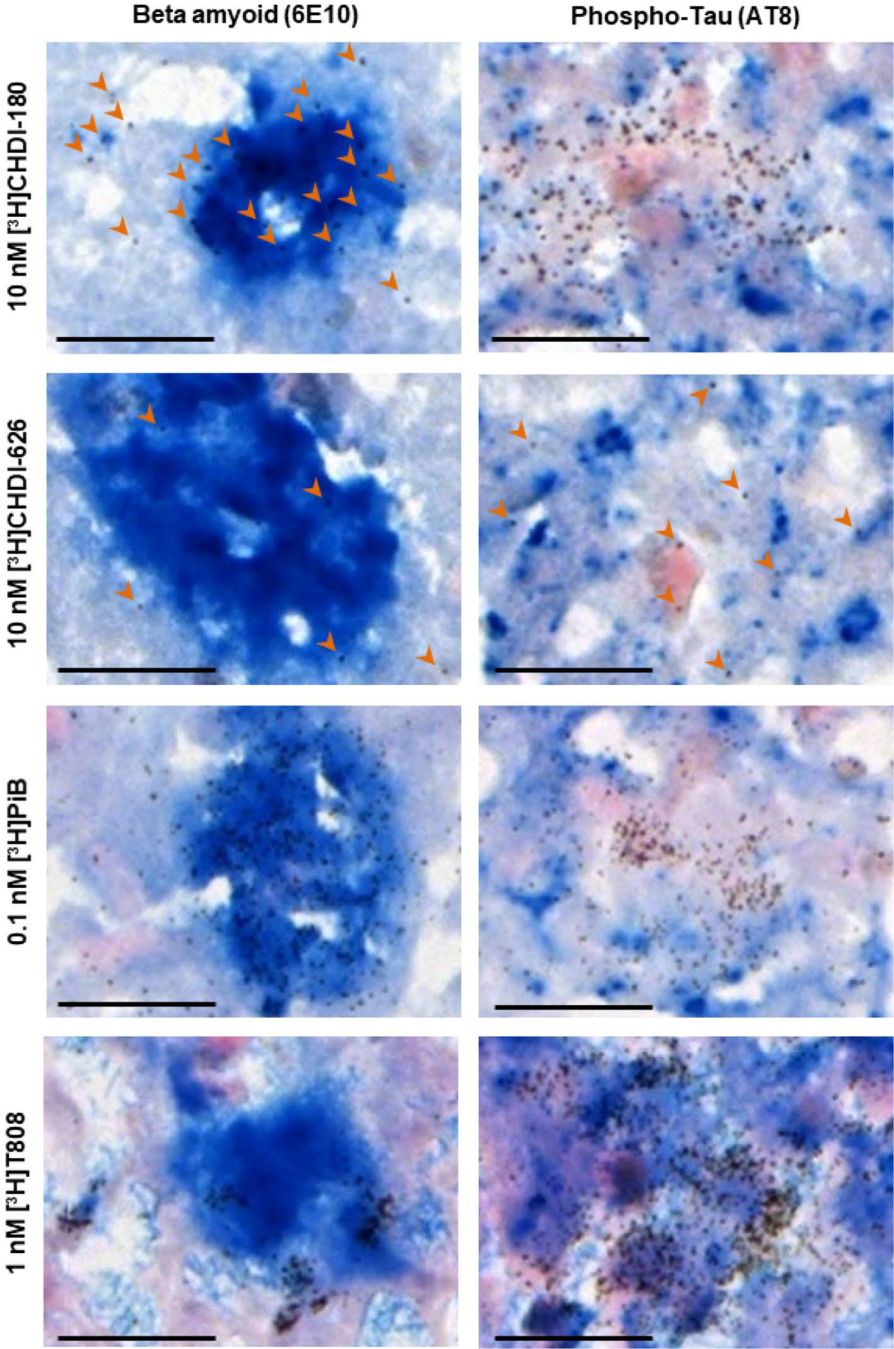
HR ARG studies investigating co-registration of radioligand binding with beta amyloid plaques and phospho-Tau tangles. HR ARG study investigating co-registration of 10 nM [^3^H]CHDI-180, 10 nM [^3^H]CHDI-626, 0.1 nM [^3^H]PiB and 1 nM [^3^H]T808 with non-HTT fibrillary deposits such as beta amyloid (6E10 antibody) or tau tangles (AT8 antibody). Bound radioligand appears as cluster of silver grains (black dots, orange arrow heads indicate individual silver grains in tissue with low levels of bound radioligand); 6E10- and AT8-positive staining is indicated as blue signal, background was stained with Nuclear Fast red and shows a pink color. Scale represents 20 µm.

As shown in Fig. 8, 10 nM [^3^H]CHDI-180 demonstrated low density binding to 6E10-positive beta amyloid plaques but did not co-register with AT8-positive phospho-Tau immunoreactivity. 10 nM [^3^H]CHDI-626 showed no co-registration with either 6E10 or AT8-positive staining signals, substantiating the results obtained in the AD brain homogenate RBA as well as standard ARG that demonstrated little-to-no binding to AD-derived material. To confirm the validity of the technique and to evaluate the magnitude of the [^3^H]CHDI-180 co-registration with 6E10 antibody, the known beta amyloid-specific tracer [^3^H]PiB (**6**) and tau-specific tracer [^3^H]T808 (**7**) were included in the study. As expected, 0.1 nM [^3^H]PiB demonstrated significant co-registration signal with 6E10 but not with AT8 immunoreactivity, whereby 1 nM [^3^H]T808 co-registered with AT8 but not with 6E10 antibody. These data confirm that the beta amyloid-plaque binding signal of [^3^H]CHDI-180 is very low compared to an established AD tracer, PiB, and that the follow-up compound, [^3^H]CHDI-626, showed improved off-target selectivity compared to [^3^H]CHDI-180.

## Discussion

We have developed a platform of in vitro*/*ex vivo assays to identify, profile and pharmacologically characterize small molecules that bind to mHTT aggregates. Our small-molecule binding protocol was adapted from the well documented filter trap or filter retardation methodology previously described for antibody-mediated immunodetection of filter-immobilized mHTT aggregates^45,56–59^. Noteworthy, however, is that these conventional paradigms were unsuccessful for radioligand binding due to unacceptable background/noise, attributed to non-specific binding of radioligands to assorted filter materials and inappropriate blocking buffers. We identified an optimized set of conditions (see Materials & Methods) that allowed medium-throughput screening of a compound library in a 96-well format which led to the discovery of several chemical scaffolds that exhibited binding to recombinant mHTT-Exon1-Q46- or Q46-derived aggregates^38,39^.

Interestingly, most novel compounds (e.g., CHDI-180 and CHDI-626; see Table 1) that were initially characterized, displayed similar pharmacology for binding to Exon1-Q46 compared to Q46, when assessing K_d_ or IC_50_. We speculate that the small molecules bind to neoepitope(s) created by the misfolding and oligomerization/aggregation of the expanded polyQ stretch expressed on both the HTT Exon1 as well as the pure expanded polyQ constructs. Although both proteins displayed similar binding properties, there are some subtle differences exhibited by each of these aggregates/fibrils, as reflected by differences in MW1 immunoreactivity, ThS fluorescence and electron microscopic analysis shown in Supplementary Fig. S2. Nevertheless, ThS fluorescence and EM morphology of these proteins are consistent with formation of fibrils that are rich in beta-sheet structures^60–71^. Noteworthy, due to the binding of our small molecules to an aggregated form of a simple polyQ-tract (e.g. Q46), it was not unexpected that CHDI-180 also bound to aggregated ataxin 1 (see Supplementary Fig. S11), another polyQ- repeat protein that plays a significant pathophysiological role in Spinocerebellar ataxia (reviewed in^72–74^). However, the ARG signals were much lower in brain sections of 2 Sca mouse models^75,76^ when compared to the R6/2 HD model; the reason for the observed ARG signal differences between the Sca and HD models is not known, but may be attributed to differences in ataxin- vs. huntingtin-aggregate affinities and/or in mouse model aggregate load expression.

One limitation of the RBA is the pore size of the filter medium, designed to entrap particles larger than the pore size (1 µm) but allow smaller proteins to go through the membrane. Indeed, when we tried to transfer our RBA, that was successful for recombinantly-derived mHTT aggregates, to assess binding of small molecules to mHTT aggregate-containing brain homogenates from mouse models of HD, a smaller binding signal was measured with homogenates from HOM zQ175 brains (compare low B_max_ from saturation binding in RBA, in Fig. 3B, to higher B_max_ observed in ARG, in Fig. 3A) and no appropriate binding signal could be generated from R6/2 brains (data not shown). This suggests that only a minor fraction of HTT target species is retained on the RBA filter plate and smaller aggregate species are potentially washed through the pores and not assayed.

To allow satisfactory signal-to-background detection using brain homogenate-derived aggregates, we adapted the filter trap assay and used a conventional filter membrane with subsequent exposure to phosphorimaging as a readout (RAFTA). The newly established RAFTA allowed the translation of our in vitro assay using recombinant aggregates to using brain-derived HTT aggregates from HD mouse models and enabled the investigation of binding properties of our small molecules to more physiological mHTT aggregate species. Despite our success using the RAFTA paradigm to interrogate small molecule binding to HD mouse model brain homogenates, we were unable to obtain satisfactory and consistent signal over background when applying this technology to human post-mortem HD brain samples (data not shown), likely due to the significantly lower density of aggregate load in human versus animal HD brain tissue.

One of the assumptions in conducting binding assays with either mHTT recombinantly-derived proteins or HD brain-derived homogenates is that the binding epitopes and/or protein conformations are retained during the generation and extraction protocols and represent those that are physiologically expressed in HD. To address this, we measured in situ binding with tritium-labeled small molecules by ARG to determine if the small molecule binding pharmacology we observed with RBA and/or RAFTA aligns to the profile obtained with mHTT aggregates in situ and expressed in HD pathology. We successfully confirmed that tritiated CHDI-180 (Fig. 4C, D and supplementary Fig. S9A) and CHDI-626 (Supplementary Figs. S8B and S9B) bind to mHTT aggregates expressed in brain sections from HOM zQ175 and R6/2 HD mouse models. Furthermore, the affinity (K_d_) of tritiated CHDI-180 and CHDI-626 were comparable in ARG experiments with zQ175 HD mouse brain slices compared to measurements using RBA performed with zQ175 brain homogenates (Fig. 3A, B and Supplementary Fig. S8A, B).

The autoradiographic analysis not only provides spatial information on binding to different regions of the brain but also to white versus gray matter. For example, there appears to be greater signal/binding in specific sub-regions compared to others in the cortical sections displayed in Figs. 4D, 6B and 7C; specifically, the areas showing greater CHDI-180 and CHDI-626 signals/binding align well with gray matter versus white matter, as revealed by using toluidine blue counterstaining (Figs. 6B, 7C). Additionally, one can detect binding specifically to the finer structure of the hippocampus, as revealed in the autoradiogram from [^3^H]CHDI-180 binding to HOM zQ175 hippocampal sections compared to the absence of such binding in age-matched WT (Fig. 4D).

As previously demonstrated^47–50^ for HD mouse models and expanded upon in this report, we observed an age- (or disease-) dependent increase in brain (hippocampus, cortex and striatum) aggregate load (density and size) in progressively increasing ages of HOM zQ175 and R6/2 mice using immunochemistry with mEM48 antibody (Fig. 5B, C); no significant immunoreactive signal was detected at any age from age-matched wild-type controls, as these mice do not express mHTT nor HTT aggregates (Supplementary Fig. S5A). Similarly, we observed an increase in [^3^H]CHDI-180 binding in this age-progressive series using our radiometric assays ARG and RAFTA (Fig. 4A–D). This suggests that the binding target for [^3^H]CHDI-180 is not unexpanded HTT but either mHTT-derived aggregates or some other state of mHTT that may be increasing in an age- and disease-progressive fashion. To address which mHTT-related target reflected the binding of CHDI-180, we took advantage of two MSD-based quantitation assays recently developed and published^44,48^; one assay interrogates mHTT (expanded polyQ; soluble mHTT)^48^ whereas the other assay reports out on aggregated mHTT levels^44^. The MSD quantitation of brain homogenates from the HOM zQ175 and R6/2 age-progression series revealed an increase in HTT aggregates (Fig. 5B, C and Supplementary Fig. S5B), which correlates well with the increase observed by IHC (mEM48 immunoreactivity) as well as ARG and RAFTA binding signals with [^3^H]CHDI-180 (Fig. 5); in contrast, the soluble expanded HTT levels actually decreased over this same age series, consistent with the hypothesis that the dynamics between the soluble and aggregated expanded HTT pools are likely shifting toward the aggregation of expanded HTT as the animal ages and the disease progresses^47–50^. Thus, our results are consistent with CHDI-180 recognizing and binding to the higher-order aggregated forms of expanded mHTT but not to the less fibrillar, more soluble expanded HTT states.

We confirmed the translatability of these mHTT aggregate binders from mouse HD model-derived tissues to humans and corroborated and expanded on our previous data showing that [^3^H]CHDI-180 exhibits mHTT-specific in situ binding in post-mortem human HD brains^39^. For CHDI-180, we showed significantly greater binding in human HD cortical than in control cortical brain tissue (Fig. 6A), However, there was even greater signal/binding in AD tissue (to either HD or CTRL signal/binding), suggesting that this compound may have some affinity for AD pathology (e.g. beta amyloid plaques and/or tangles). This was not surprising, since mHTT can form a beta-sheet signature like amyloid fibrils and [^3^H]CHDI-180 was derived from known PET tracers targeting beta amyloid plaques. However, when compared to known beta amyloid/tau binders, like PiB and T808, the binding level of [^3^H]CHDI-180 in AD brain section is minimal (Fig. 6A and Supplementary Fig. S6A, B).

We established a high-resolution microscopic emulsion autoradiography assay (HR ARG), a combination of radioligand binding and recognition of the target structure at the cellular level by immunohistochemistry, to gain more knowledge about the specificity (AD vs. HD binding) and non-specific vs. off-target binding of CHDI-180 binding regarding aggregates derived from other misfolded, amyloid, β-sheet-forming proteins, like beta amyloid and tau^77–81^ in situ. Interestingly, [^3^H]CHDI-180 demonstrated some binding to 6E10-positive beta amyloid plaques but did not co-register with AT8-positive phospho-Tau tangles (Fig. 8). These data suggest that the observed binding of CHDI-180 to Alzheimer’s disease brains is due to beta amyloid-plaque binding properties of the small molecule but is very low compared to PiB as an established beta amyloid directed tracer.

These results indicate that further medicinal chemistry efforts in understanding structure–function relationships of binding to both AD-associated and HD-associated aggregates could lead to more selective HTT aggregate-specific small molecule binders. Such selectivity for disease-specific pathology (i.e. mHTT vs. beta amyloid/tau aggregates) is highly desirable in designing and developing an HTT-aggregate-directed PET tracer to minimize any signal confound attributed to co-morbidity (i.e. presence of beta amyloid pathology) in HD patients. On the other hand, lack of selectivity and the availability of a small molecule binder to a common epitope shared among more than one misfolded, aggregated protein (e.g. HTT and ataxin or other proteins that possess beta-sheet conformations^82–84^) could provide an opportunity to develop a multi-indication PET tracer that recognizes other expanded polyQ proteins (e.g. ataxin for Sca; see Supplementary Fig. S11) or therapeutics.

As we continued our adaptation of known beta-sheet binders from the Alzheimer’s field, we found that inclusion of the BIP tricyclic core from T808 resulted in a new chemotype^38^ with potent binding affinity for recombinant mHTT-derived aggregates (Table 1) and good specific binding in mouse models of HD (Supplementary Fig. S8 and S9B), but a significantly lower AD liability in human tissue than CHDI-180 (Fig. 7A–C). The new compound CHDI-626 has improved off-target selectivity in AD brain tissue compared to CHDI-180 as shown by the lower binding affinity in the ADbh RBA (Supplementary Fig. S7), with a maximum inhibition of only 5% at 1 µM (Fig. 7A) and in human post-mortem brain sections from HD, AD and CTRL subjects using in situ autoradiography (Fig. 7B, C) and HR ARG (Fig. 8).

In conclusion, by using a series of newly developed or adapted binding assays, we were able to identify and subsequently characterize small molecule binders of mHTT aggregates. The assays utilizing recombinantly-derived mHTT provided robust absolute and relative potencies of binding activities of our small molecules and in addition allowed further understanding of the structure–activity relationships of several chemotypes which led to the identification of binders of mHTT aggregates. It is important to note that the initial assays utilizing recombinant HTT-derived aggregates aligned well with more physiologically relevant biological material, which we addressed using HD animal brain homogenate-derived material as well as in situ bindings by autoradiography.

We aim to utilize this portfolio of assay to design and test more potent and selective (against other amylogenic proteins) small molecule HTT aggregate binders in medicinal chemistry SAR campaigns. The identification of potent and selective small molecule binders of HTT aggregates as well as other states of mHTT (i.e., soluble/monomers) will enable the development of additional imaging tools (i.e. alternative epitopes) and may open up new therapeutic avenues, such as small molecule disaggregators or protein degraders (e.g. PROTACs, AUTACs and LYTACs) to promote mHTT clearance/degradation.

## Materials and methods

### Radioligands

Isotope-labeled ([^3^H]) radioligands were customer-synthesized (except for **6**) as ethanol stocks. The following radioligands, with the indicated specific activities, stock concentrations and vendors (in parentheses), were utilized (for structures, see Supplementary Fig. S1):

**1** (CHDI-180) with 79 Ci/mmol at 12.6 µM (Novandi), **2** (CHDI-626) with 81 Ci/mmol at 12.3 µM (Novandi), **3** with 82 Ci/mmol at 12.1 µM (Pharmaron), **4** with 69 Ci/mmol at 14.4 µM (Novandi), **5** with 80 Ci/mmol at 12.5 µM (Novandi), **6** (PiB) with 76 Ci/mmol at 3.2 µM (ViTrax, VT 278), **7** (T808) with 63 Ci/mmol at 15.8 µM (Pharmaron) and **8** with 27.8 Ci/mmol at at 35.9 µM (Moravek).

### Recombinant poly-glutamine proteins

Two different poly-glutamine(Q) proteins were generated, each containing a cleavable amino-terminal tag to prevent aggregation during expression and purification: GST-Q46 (termed “Q46” in future context) with amino acid sequence MSPILGYWKI KGLVQPTRLL LEYLEEKYEE HLYERDEGDK WRNKKFELGL EFPNLPYYIDG DVKLTQSMAI IRYIADKHNM LGGCPKERAE ISMLEGAVLD IRYGVSRIAY SKDFETLKVD FLSKLPEMLK MFEDRLCHKT YLNGDHVTHPD FMLYDALDVV LYMDPMCLDAF PKLVCFKKRI EAIPQIDKYLK SSKYIAWPLQG WQATFGGGDH PPKSDLVPR (thrombin cleavage site) G SQQQQQQQQQ QQQQQQQQQQ QQQQQQQQQQ QQQQQQQQQQQ QQQQQQ and MBP-HTT(1-89)Q46-His(6x) (termed “Exon1-Q46” in future context) with amino acid sequence MGKIEEGKLV IWINGDKGYN GLAEVGKKFE KDTGIKVTVE HPDKLEEKFP QVAATGDGPD IIFWAHDRFG GYAQSGLLAE ITPDKAFQDK LYPFTWDAVR YNGKLIAYPI AVEALSLIYN KDLLPNPPKT WEEIPALDKE LKAKGKSALM FNLQEPYFTW PLIAADGGYA FKYENGKYDI KDVGVDNAGA KAGLTFLVDL IKNKHMNADT DYSIAEAAFN KGETAMTING PWAWSNIDTS KVNYGVTVLP TFKGQPSKPF VGVLSAGINA ASPNKELAKE FLENYLLTDE GLEAVNKDKP LGAVALKSYE EELAKDPRIA ATMENAQKGE IMPNIPQM SAFWYAVRTA VINAASGRQT VDEALKDAQT NSSSNNNNNN NNNNLGENLY FQ (TEV cleavage site) GSLVPR (thrombin cleavage site) GG MATLEKLMKA FESLKSFQQQ QQQQQQQQQQ QQQQQQQQQQ QQQQQQQQQQ QQQQQQQQQQ QQQPPPPPPP PPPPQLPQPP PQAQPLLPQP QPPPPPPPPP PGPAVAEEPL HRHHHHHH (Supplementary Fig. S2A).

For bacterial production, the cDNA sequences encoding the HTT proteins were codon-optimized; GST-Q46 and MBP-HTT(1-89)Q46-His(6x) were cloned into pGEX4T1 (GenBank MT364377) and pTrilJ-MV (GenBank MT350567) expression vectors, respectively. Protein expression and purification protocols are described in the supplementary materials and methods section.

### Protein aggregation

For N-terminal tag cleavage, proteins at 30 µM in 2 mL reaction tubes were incubated with 150 µg/mL thrombin (Sigma, T4648) in 50 mM Tris (pH 8.0), 150 mM NaCl, 2 mM CaCl_2_ for 16 h at 37 °C. For both Q46 and Exon1-Q46, removal of GST or MBP, respectively, initiated the misfolding, nucleation and elongation processes and resulted in the formation of aggregated multimers (referred to as aggregates)^57^. We had previously demonstrated that aggregation followed a time course with maximal binding plateauing at > 8 h post-N-terminal cleavage and utilized 16 h for practical reasons.

For radioligand binding assays (RBA) (see below) and radiometric filter trap assays (RAFTA) (see below), aggregates were separated by centrifugation (5 min 16,000×g) and resuspended with assay buffer (50 mM Tris pH 8.0, 150 mM NaCl) (see Supplementary Fig. S2B). Protein concentrations always refer to the starting peptide concentrations (i.e., peptide concentration applied in the aggregation reaction before thrombin cleavage) since post-cleaved aggregates are heterogeneous and the number of peptides per aggregate is unknown.

For ARG and immunohistochemistry (IHC) analyses, fresh frozen whole brain samples from zQ175 and R6/2 and age-matched wild-type (WT) mice were prepared (Table 2). The mice were either euthanized by cervical dislocation or PBS-perfusion and the brains were frozen in isopentane at − 30 to − 40 °C and stored at − 80° C. Animal handling and all subsequent procedures were carried out in accordance with the regulations of the German animal welfare act and the EU legislation (EU directive 2010/63/EU) and were approved by the Authority for Health and Consumer Protection of the city and state of Hamburg (“Behörde für Gesundheit und Verbraucherschutz” BGV, Hamburg). This study was carried out in compliance with the ARRIVE guidelines^85^.

**Table 2:** Mouse strain information.

Common name	Strain name/standardized nomeclature	Repeat length/allele type	Gene characteristics	Provider
zQ175DN	B6J.129S1-Htttm1.1Mtc/190ChdiJ	180–220 CAG/knock-in	Endogenous murine Htt gene, chimeric human/mouse exon 1	CHDI
R6/2	B6CBA-Tg(HDexon1)62Gpb/125JChdi	128 CAG/Tg fragment	HTT promoter, exon 1 of human HTT	CHDI
PcP2-82Q		82 CAG/Tg	Human ataxin-1 gene with expanded CAG under control of the purkinje cell-specific PcP2 promoter	Dr. Harry Orr (University of Minnesota)
SCA154Q/2Q		154 CAG/knock-in	Endogenous murine Sca1 locus	Dr. Huda Zoghbi (Baylor College of Medicine)

### Human brain tissue: procurement and QC by immunohistochemistry (IHC)

Frontal cortical post-mortem brain tissue from HD patients, controls (CTRL) without any evidence of neurological disease, and Alzheimer´s Disease (AD) patients were obtained from New York Brain Bank (NYBB), Netherland Brain Bank (NBB) and Tissue Solutions Ltd. A summary of the demographic characteristics of the cases studied is shown in Supplementary Table S1.

Prior to use, all human brain samples underwent quality assessment by IHC analysis. Expression of mHTT aggregates was confirmed by mEM48 (Merck Millipore; MAB5374) IHC in HD brains. Furthermore, all brain tissues were assessed for the presence of beta amyloid by 6E10 (BioLegend, #803003) IHC and presence of phosphorylated tau by AT8 (ThermoFisher Scientific, MN1020) IHC. For both mHTT aggregate and beta amyloid as well as tau evaluation, 10 µm-thick sections were prepared from frozen frontal cortex (CTX) tissue blocks by using a cryostat, mounted on superfrost slides and stored at − 80 °C for a maximum of 2 weeks. The sections were post-fixed with 4% paraformaldehyde for 10 min followed by two times washing with TBS (Tris buffered saline, pH 7.4). Epitope retrieval was done with 1% formic acid for 10 min followed by washing with TBS. Non-specific binding sites were blocked for 20 min with 2.5% horse serum (Vector Laboratories). Sections were incubated for 1 h with mEM48 (1:500), 6E10 (1:800) or AT8 (1:500) antibodies at room temperature followed by a washing step in TBS.

The ImmPress-AP anti-mouse IgG polymer detection kit (Vector Laboratories, MP-5402) and Vector blue alkaline phosphatase substrate kit (Vector Laboratories, SK-5300) were used as the detection system according to the manufacturer’s instructions. Sections were treated for 10 min with Nuclear fast red (Vector Laboratories, H-3403–500) for nuclear counterstain. Only beta amyloid-negative and phosphorylated tau-negative HD and CTRL brain samples were included in the studies that focused on mHTT aggregate binding. Representative images are shown in Supplementary Fig. S3.

### Preparation of brain homogenates for radioligand binding assays (RBA), radiometric filter trap assay (RAFTA) and meso scale discovery (MSD) analysis of HTT expression

For RBA in vitro binding assays, brain tissue was sliced (50 µm), weighed and homogenized in PBS (Gibco, 14190-94), 0.1% BSA with a glass pestle (10 strokes) at a concentration of 12–15 mg wet tissue per mL buffer. The crude homogenate concentration was then adjusted to 10 mg/mL (wet tissue) and stored as single-use aliquots at -80 °C.

For RAFTA and MSD assays, mouse brains were transferred to lysis tubes and individually homogenized 1:3 (w/v) in brain lysis buffer (10 mM Tris pH 7.4, 800 mM NaCl, 1 mM EDTA, 1 × Protease inhibitor, 10% Sucrose, 0.5% Triton X-100, Benzonase (1:1000)) using a pre-cooled MP FastPrep homogenizer (3 × 30 s, 6.0 m/sec). Crude homogenates were transferred to LoBind reaction tubes (Eppendorf) and centrifuged at 15,700×g for 10 min at 4 °C. The supernatant was collected, and the pellet fraction was resuspended in brain lysis buffer. Generally, a minimum of three whole brains were lysed and fractions were pooled after homogenization to reduce assay variability due to inter-animal variability.

The total protein concentration of samples was determined by BCA assay (ThermoFisher Scientific, #23227), according to standard procedures and protein concentrations were adjusted to 5–10 mg/mL in brain lysis buffer. The adjusted homogenate fractions were divided into single-use aliquots, snap-frozen on dry ice and stored at -80 °C.

### Radioligand binding assay (RBA)

For the competition assay format, Q46 or Exon1-Q46 aggregates at 1 µM (sample volume 150 µL) were pre-incubated in a 96-well round bottom plate (Corning, #3365) for 20 min with test compounds at room temperature (assay buffer 50 mM Tris–HCl pH 8.0, 150 mM NaCl). Test compounds were serially diluted in DMSO and transferred directly into the assay without aqueous dilution; final DMSO concentration in the assay was 3%, previously shown not to significantly affect binding performance (data not shown). Radioligand **3** at 0.3 nM was added and incubated for 1 h at 37 °C.

Samples were then transferred onto a 96-well GF/B filter plate (Perkin Elmer, #6005177) using a Filtermate Harvester, aspirated by vacuum and wells washed twice with 200 µL PBS. After drying the filter plates for 1 h at 55 °C the back of the plate was sealed with aluminum cover and 30 µL of scintillation fluid (Perkin Elmer, #6013641) per well was added. The tops of the plates were sealed, and the plates were incubated for 15 min in the dark and counted in a TopCount or MicroBeta2 reader (both Perkin Elmer).

Raw sample data collected as counts per minute (cpm) were normalized towards assay controls: 0% inhibition = radioligand + protein + vehicle, 100% inhibition = radioligand + protein + 1 µM unlabeled ligand. A 4-parameter hyperbolic fit was applied to generate IC_50_ values.

For saturation binding experiments, aggregate lysate preparation and final concentration in the assay, assay buffer, incubation times and plate processing were identical to the competition format. Radioligand was titrated up to 100 nM. Raw cpm were converted into nM using a calibration line of total added radioligand without washing and filtering (spiked onto the filter plate and dried) (e.g., X) and normalized to the concentration of protein (e.g., Y) to finally obtain nmol/µmol (e.g., X/Y). Specific binding (SB) was calculated as full binding minus full competition (in the presence of excess unlabeled ligand) and a hyperbolic mono-phasic fit was applied using GraphPad Prism to determine K_d_ and B_max_.

RBA experiments using Alzheimer’s disease brain homogenates (ADbh RBA) followed the same assay principle as the RBA with recombinant HTT protein, using 0.68 mg/mL homogenate (100 µg per well with 150 µL assay volume) in assay buffer PBS, 0.01% Pluronic F127 (Sigma. P2443) and 1% DMSO after compound addition. Competition experiments were conducted with 0.5 nM radioligand **4** and 50 µg homogenate per well. Incubation at room temperature was conducted for 20 min after compound addition and for 5 h after radioligand addition. For the competition format, raw cpm were normalized towards assay controls as before, to obtain inhibition between 0 and 100%. For saturation binding, raw cpm were converted into nM as before, using a calibration line of total added radioligand, and normalized to mg of added homogenate to finally obtain pmol/mg.

### Radiometric filter trap assay (RAFTA)

For RAFTA analysis, frozen mouse brain homogenates were thawed on the day of the experiment and diluted with assay buffer (50 mM Tris pH 8.0; 150 mM NaCl) to a concentration of 500 µg/mL total protein. For saturation binding experiments, radioligand CHDI-180 (**1**) was diluted in assay buffer (0–10 nM final conc.) and 100 µL of diluted radioligand were mixed with 100 µL homogenate and incubated for 1 h at 37 °C without shaking. In experiments where non-specific binding (NSB) was determined, the incubation medium contained 5 µM of non-labeled (cold) CHDI-180, which was added prior to the addition of radioligand and incubated for 20 min at room temperature.

A GF/F membrane (Whatman, 1825–293) was pre-wetted in PBS and inserted into a Bio-Dot Microfiltration Apparatus (Bio-Rad). After blocking the membrane by filtration of 1% Pluronic / 3% BSA (3 × 200 µL/well) and washing two times with 200 µL PBS, each sample was vacuum-filtered through the membrane to remove unbound radioligand. After washing the membrane twice with 200 µL PBS the membrane was removed and dried at 55 °C for 1 h.

After drying, the membrane and a tritium microscale standard (e.g. ART0123B(PL)-1EA, ARC Inc.) were placed into an X-ray cassette and exposed to a TritiumPhosphor Screen (GE Healthcare; Fuji BAS-IP TR 2025 E) for 4–7 days followed by quantitation of bound radioactivity using a Phosphorimager (Typhoon FLA 7000) and ImageQuant 8.0 TL Software (both GE Healthcare).

Raw counts (relative fluorescence units or RFUs) were converted into fmol/mg tissue using the tritium microscale standard as reference. SB was determined by subtracting NSB. Data were fitted to one-site binding equation using a non-linear regression method in GraphPad Prism Software.

### Autoradiography (ARG)

Twenty µm-thick sections were prepared from brains of disease mouse models and WT mice or human post-mortem brain samples by using a cryostat, mounted on superfrost slides and stored at -80 °C for a maximum of 2 weeks. On the day of the experiment, slides were adapted to room temperature for 30 min and then equilibrated by immersion into assay buffer (50 mM Tris–HCl pH 7.4; 120 mM NaCl; 5 mM KCl; 2 mM CaCl_2_; 1 mM MgCl_2_) for 20 min at room temperature.

Radioligand solutions were prepared in assay buffer. Optimal radioligand concentrations were determined in advance based on signal-to-background ratio obtained in mouse or human brain tissue. The solutions were mixed in a Coplin jar (Sigma, S5516-6EA) by gently shaking at 175 rpm for 10 min at room temperature. Sections were incubated by immersion into assay buffer containing either only radioligand (total binding or TB) or radioligand plus 10 µM of unlabeled compound (non-specific binding or NSB) for 60 min at room temperature. Afterwards slides were washed three times for 10 min with ice-cold washing buffer (50 mM Tris–HCl (pH 7.4)) at 4 °C and dipped for three seconds in ice-cold distilled water to remove buffer salts. The slides were dried for 3 h at 30 °C and exposed for 96 h to a Tritium Phosphor Screen (GE Healthcare, Fuji BAS-TR 2025 E) Stored radiation energy on the screen was scanned using a Phosphorimager (GE Healthcare, Typhoon FLA 7000). Densitometric data analysis of radioligand binding was performed using the MCID Analysis 7.1 software (Interfocus Imaging Ltd.). Radioisotope concentration standards (American Radiolabeled Chemicals, ART 0123C and ART 0123B) were exposed with each specimen to enable conversion of density values into tissue-equivalent ligand concentrations (e.g., fmol/mg tissue). A standard curve was created to relate the system´s internal relative optical density measurement (ROD; an inverse logarithmic function of gray level values) to the set of standard values (e.g., isotope concentration). The standard´s isotope concentration (e.g., nCi/mg) is therefore converted into tissue-equivalent ligand concentrations (e.g., fmol/mg tissue) by referring to the radioligand´s specific activity (e.g., Ci/mmol) and including decay-correction. After phosphor screen exposure the human brain sections were stained for 4 min with 0.1% toluidine blue dye solution (Sigma-Aldrich) and rinsed twice in distilled water. To differentiate gray and white matter, the slides were decolorized with 70% ethanol (Merck) for 2–5 min followed by rinse in distilled water. Gray matter appears as gray color and white matter as bluish.

### High resolution autoradiography (HR ARG): co-registration studies

Ten µm-thick sections were prepared from human post-mortem brain samples by using a cryostat, mounted on superfrost slides and stored at -80 °C for a maximum of 2 weeks. On the day of the experiment, slides were adapted to room temperature for 30 min. The sections were post-fixed with 4% paraformaldehyde for 10 min followed by two times washing with TBS (20 mM Tris–HCl, pH 7.4, 150 mM NaCl). Epitope retrieval was done with 1% formic acid for 10 min followed by washing with TBS.

Slides were equilibrated by immersion into autoradiography assay buffer (50 mM Tris–HCl pH 7.4; 120 mM NaCl; 5 mM KCl; 2 mM CaCl_2_; 1 mM MgCl_2_) for 20 min at room temperature. Radioligand solutions were prepared in assay buffer. Optimal radioligand concentrations were determined in advance based on signal-to-background ratio obtained in human brain tissue. The solutions were mixed in a Coplin jar (Sigma, S5516-6EA) by gently shaking at 175 rpm for 10 min at room temperature. Sections were incubated by immersion into assay buffer containing either only radioligand (TB) or radioligand plus excess concentration of unlabeled compound (NSB) for 60 min at room temperature. Afterwards slides were washed three times for 10 min with ice-cold washing buffer (50 mM Tris–HCl, pH 7.4) at 4 °C**.**

Afterwards the slides were washed with permeabilization buffer (20 mM Tris–HCl, pH 7.4), 150 mM NaCl, 0.1% Triton X-100) followed by a brief rinse with TBS. Non-specific binding sites were blocked for 1 h with Mouse on Mouse (M.O.M.) Blocking reagent (Vector Laboratories; MKB-2213) followed by washing with TBS. An additional protein blocking step was done for 20 min with 2.5% horse serum (Fitzgerald; 88R-1020). Sections were incubated for 1 h with 6E10 (1:800; BioLegend, #803001) or AT8 (1:500, ThermoFisher Scientific, MN1020) antibodies at room temperature in TBS containing 0.1% TritonX-100 (Sigma Aldrich, T9284) and 1% horse serum followed by a washing step in TBS. The ImmPress-AP anti-mouse IgG polymer detection kit (Vector Laboratories, MP-5402) and Vector blue alkaline phosphatase substrate kit (Vector Laboratories, SK-5300) were used as the detection system according to the manufacturer’s instructions. Sections were treated for 10 min with Nuclear fast red (Vectorstain, H3403) for nuclear counterstain followed by rinsing in distilled water. The slides were allowed to dry and were then covered with NTB emulsion (Kodak/Carestream, 8895666). After drying overnight, the slides were exposed for three weeks at 4 °C under light proof conditions. Photographic development was done by applying the developer X-tol (Kodak; KODAK008) and Vario Fix Powder (Tetenal, S32138) according to the manufacturer´s instructions. Slides were washed with distilled water, allowed to dry for a minimum of 5 h and covered in Poly-Mount Mounting Media (Polysciences Europe, 08381-120). Image analysis was done using the PreciPoint M8-S microscope with a 40 × air objective.

## Supplementary Information

Supplementary Information.

## Data Availability

All data generated or analyzed during this study are included in this published article (and its Supplementary Information files). The details and sequences of the expression vector plasmids, GST-Q46 (in pGEX4T1) and MBP-HTT(1-89)Q46-His(6x) (in pTrilJ-MV), are deposited at GenBank and have the accession numbers, MT364377 and MT350567, respectively. Requests for materials should be addressed to J.A.B.
