# Supplementary material for: Pharmacological characterization of mutant huntingtin aggregate-directed PET imaging tracer candidates
Source: Sci Rep. 2021 Sep 9;11:17977. doi: 10.1038/s41598-021-97334-z (PMC8429736; doi:10.1038/s41598-021-97334-z)
Supplement: Supplementary file 1 — Supplementary Information. [file 41598_2021_97334_MOESM1_ESM.docx]

**Supplementary Information**

**Pharmacological Characterization of Mutant Huntingtin Aggregate-Directed PET Imaging Tracer Candidates**

Frank Herrmann^✝1^, Manuela Hessmann^✝1^, Sabine Schaertl^✝1^, Karola Berg-Rosseburg^1^, Christopher J Brown^2^, Galina Bursow^1^, Anass Chiki^3^, Andreas Ebneth^1^, Miriam Gehrmann^1^, Nicole Hoeschen^1^, Madlen Hotze^1^, Stefanie Jahn^1^, Peter D Johnson^2^, Vinod Khetarpal^4^, Alex Kiselyov^4^, Karsten Kottig^1^, Stefanie Ladewig^1^, Hilal Lashuel^3^, Sven Letschert^1^, Matthew R Mills^2^, Kathrin Petersen^1^, Michael E Prime^2^, Christoph Scheich^1^, Gerhard Schmiedel^1^, John Wityak^4^, Longbin Liu^4^, Celia Dominguez^4^, Ignacio Muñoz-Sanjuán^4^, and Jonathan A Bard^*4^

^✝^ Authors contributed equally

^*^ Corresponding author: [Jonathan.Bard@CHDIFoundation.org](mailto:Jonathan.Bard@CHDIFoundation.org)

**Table of Contents**

| **Page** | **Item** | **Content** |
| --- | --- | --- |
| 3 | **Figure S1** | Structures of mHTT aggregate ligands |
| 4 | **Figure S2** | Exon1-Q46 and Q46 characterization |
| 5 | **Figure S3** | Representative images of mEM48, 6E10 and AT8 immunohistochemistry in human HD, CTRL and AD frontal cortex brain sections |
| 6 | **Figure S4** | Radiometric Filter Trap Assay (RAFTA) |
| 7 | **Figure S5** | Characterization of aggregate load in brains from 3-, 6-, 9- and 12-month-old HOM zQ175 mice |
| 8 | **Figure S6** | Autoradiographic studies investigating PiB and T808 ligand binding to human AD frontal cortex sections |
| 9 | **Figure S7** | AD brain homogenate RBA |
| 10 | **Figure S8** | Saturation binding of [3H]CHDI-626 using HOM zQ175 (12 months) HD mouse brain |
| 11 | **Figure S9** | In vitro ARG showing an age-dependent increase in [^3^H]CHDI-180 and [^3^H]CHDI-626 binding to brain sections of R6/2 mice |
| 12 | **Figure S10** | Autoradiographic studies of compound 4 to human AD brain sections |
| 13 | **Figure S11** | Autoradiographic studies investigating CHDI-180 ligand binding in Sca1 mouse models |
| 14 | **Table S1** | Demographic information for human HD, AD and control brains |
| 15-16 | **Table S2** | Autoradiographic summary of [3H]CHDI-180 binding in HOM zQ175 and WT brain sections. |
| 17-23 | **Supplementary Material and Methods** | Supplementary Material and Methods |


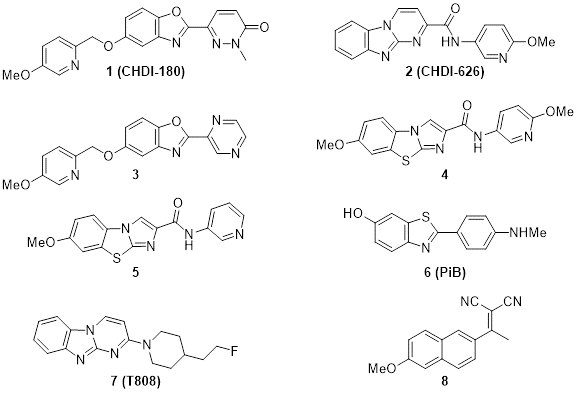


**Figure S1**: Structures of mHTT aggregate ligands.


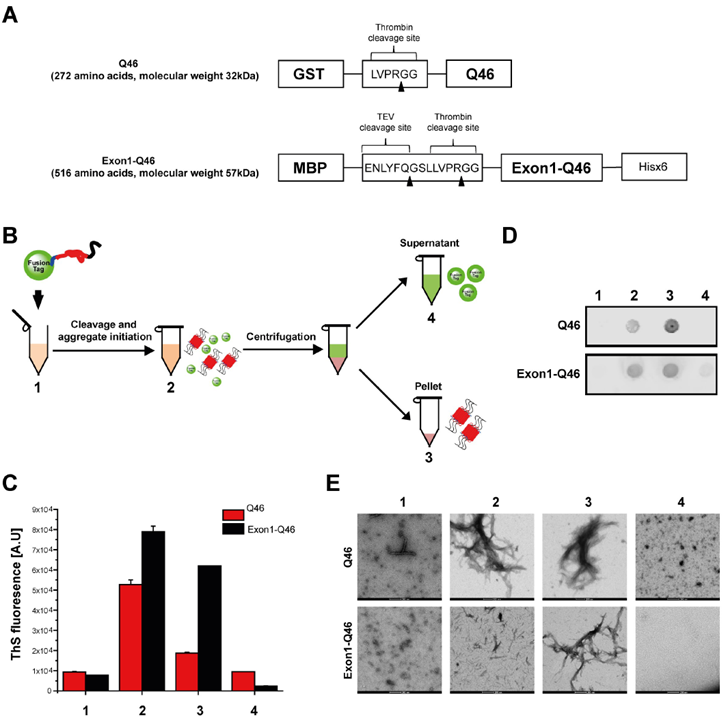


**Figure S2**: Exon1-Q46 and Q46 characterization. **A**) Schematic depiction of the procedure used to prepare and isolate Exon1-Q46 and Q46 aggregates. The material in the different fractions (numbered 1-4 in (**B**)) were characterized by ThS fluorescence assay (**C**), filter-trap immunoblot assay (**D**), and transmission electron microscopy (**E**).


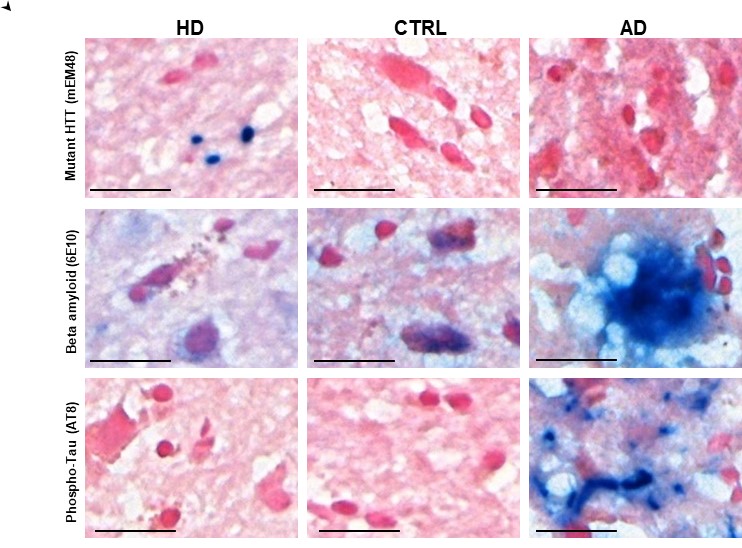


**Figure S3**: Representative images of mEM48, 6E10 and AT8 immunohistochemistry in human HD, CTRL and AD frontal cortex brain sections. Vector blue substrate was used to visualize antibody binding, Nuclear Fast Red (pink) served as counterstain. Scale represents 20 µm.


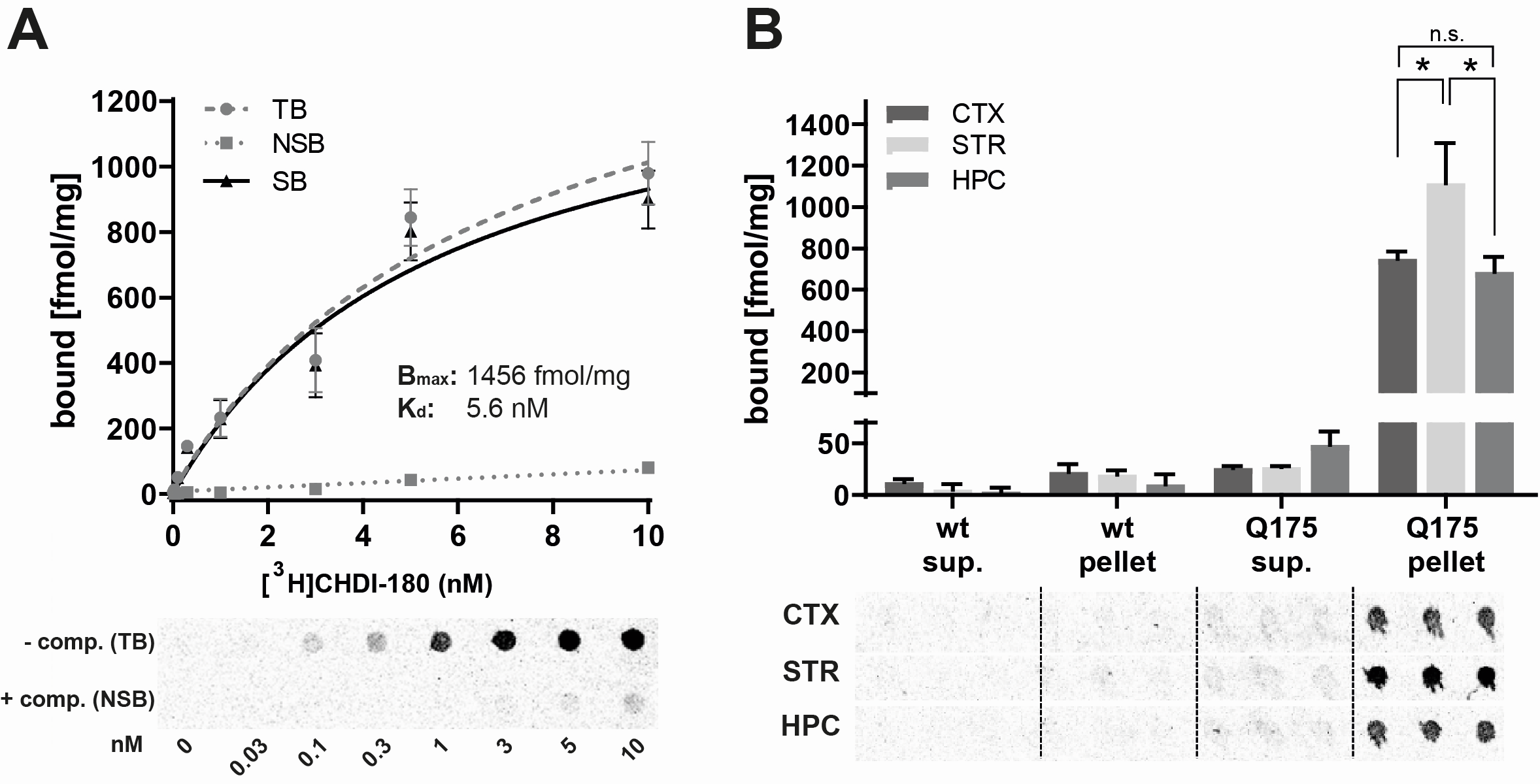


**Figure S4**: Radiometric Filter Trap Assay (RAFTA). (**A**) Saturation binding of [^3^H]CHDI-180 to determine maximal binding (Bmax) and binding affinity (Kd) values for Exon1-Q46 aggregates. Representative graphs showing total binding (TB), non-specific binding (NSB) and specific binding (SB). Mean ± SEM of 2 experiments. (**B**) [^3^H]CHDI-180 binding to HTT aggregates in centrifuged pellets, but not supernatants, of heterozygous zQ175 brain homogenates from the cortex (CTX), striatum (STR) and hippocampus (HPC). Statistical analyses were performed by Two-way ANOVA followed by Tukey's multiple comparisons test was performed. * p < 0.0001 for comparison of brain regions in zQ175 pellet fraction.


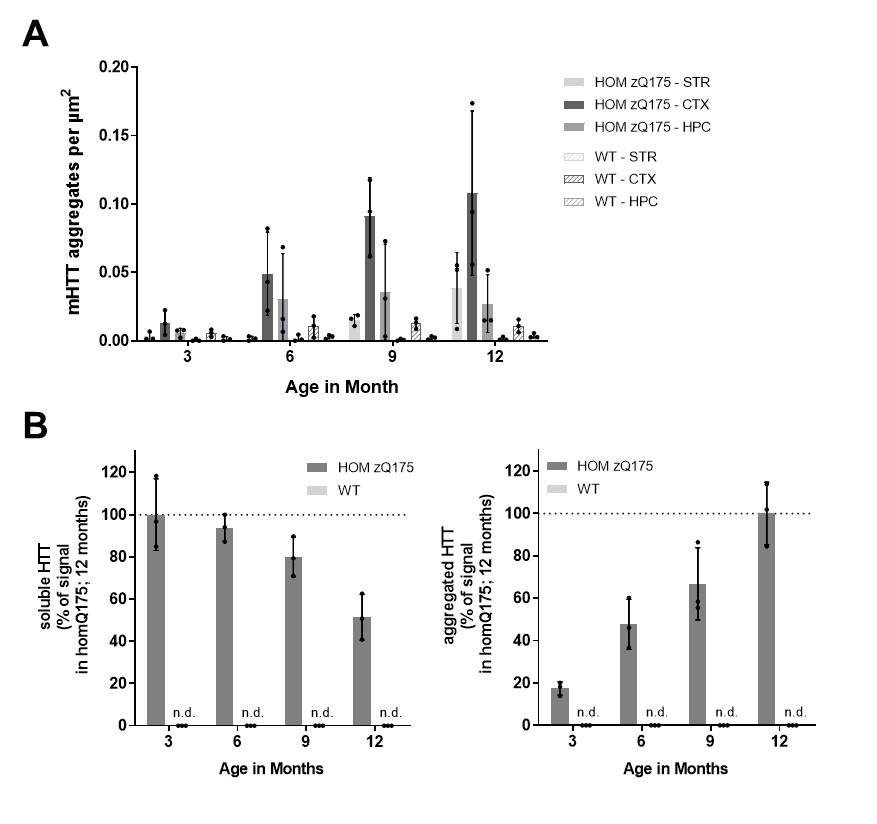


**Figure S5**: Characterization of aggregate load in brains from 3-, 6-, 9- and 12-month-old HOM zQ175 mice. (**A**) Quantification of mEM48 inclusions in STR, CTX and HPC by immunohistochemistry (IHC). Brain sections of HOM zQ175 and WT mice up to 12 months of age were subjected to immunohistochemical staining with mEM48, followed by the analysis of HTT inclusion density. (**B**) Quantitation of aggregated and soluble mHTT levels by MSD analysis. MSD data are depicted as mean ± SD from a total of n=3 brain hemispheres per age and genotype. n.d. = not detectable


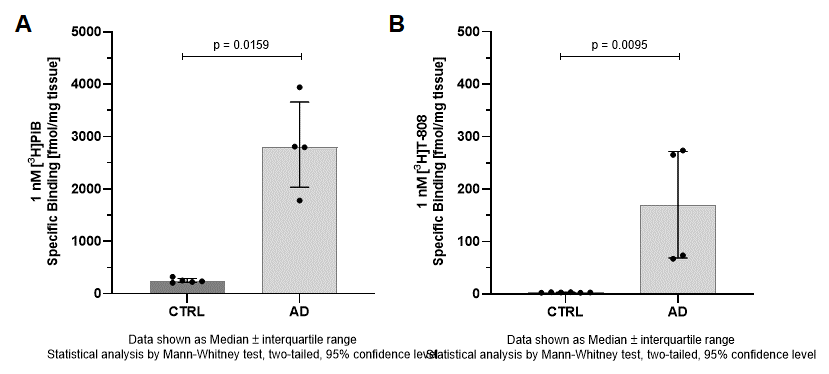


**Figure S6**: Autoradiographic studies investigating PiB and T808 ligand binding to human AD frontal cortex sections. Specific binding (SB) of **(A)** [^3^H]-PiB and **(B)** [^3^H]-T808 to human AD brain sections in *ex vivo* ARG. Data shown as median ± interquartile range. Statistical analysis by Mann-Whitney test, two-tailed, 95% confidence level.


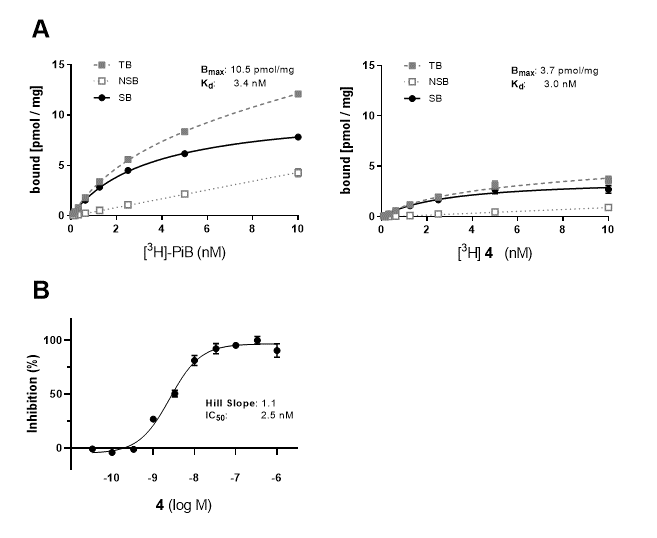


**Figure S7**: AD brain homogenate RBA (**A**) Direct binding of radioligands PiB and **4** to 100 µg of human post-mortem AD brain homogenate per well; total binding (TB) and non-specific binding (NSB) in the presence of 1 µM respective cold compound, specific binding (SB) calculated from TB – NSB; note different scales of y-axis. (**B**) Competition format to determine IC_50_ of cold **4** with 0.5nM of radiolabeled **4**.


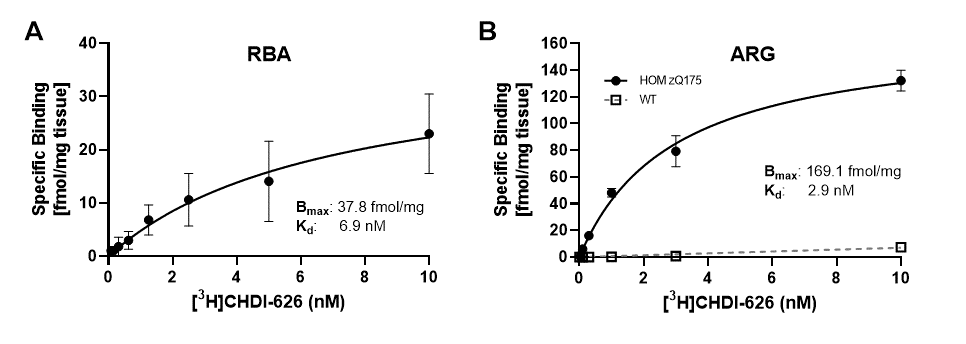


**Figure S8**: Saturation binding of [^3^H]CHDI-626 using HOM zQ175 (12 months) HD mouse brain. (**A**) Homogenate RBA. (**B**) *in vitro* autoradiography (ARG) using brain sections. Specific binding normalized per mg tissue.


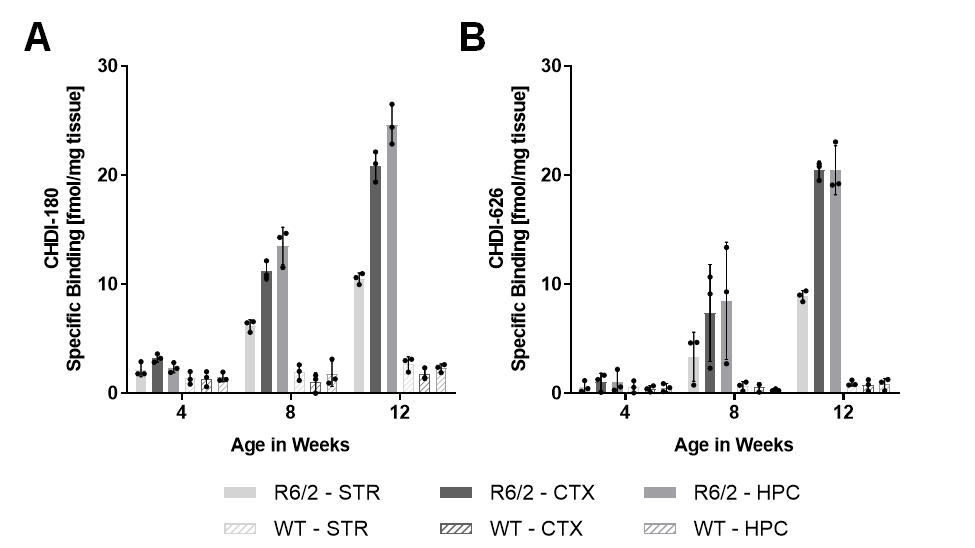


**Figure S9**: In vitro ARG showing an age-dependent increase to brain sections of R6/2 HD mice. Specific binding (SB) of (**A**) 0.5 nM [^3^H]CHDI-180 or (**B**) 1 nM [^3^H]CHDI-626 in striatum (STR), cortex (CTX) and hippocampus (HPC) of 4-, 8- and 12-week-old R6/2 mice and age-matched WT mice. Data are depicted as mean ± SD from a total of n=3 brain hemispheres per age and genotype.**
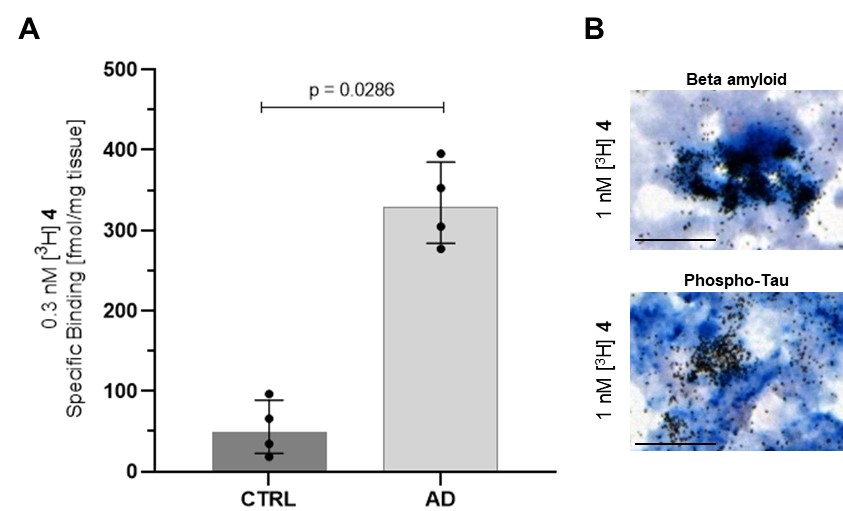
**

**Figure S10**: Autoradiographic studies of compound **4** to human AD brain sections. **(A)** ARG studies investigating specific binding (SB) of **4** (0.3 nM) to human AD frontal cortex sections. Data shown as Median ± interquartile range. Statistical analysis by Mann-Whitney test, two-tailed, 95% confidence level. **(B)** Representative images of **4** co-registration with 6E10 but not AT8 in human AD brain sections.


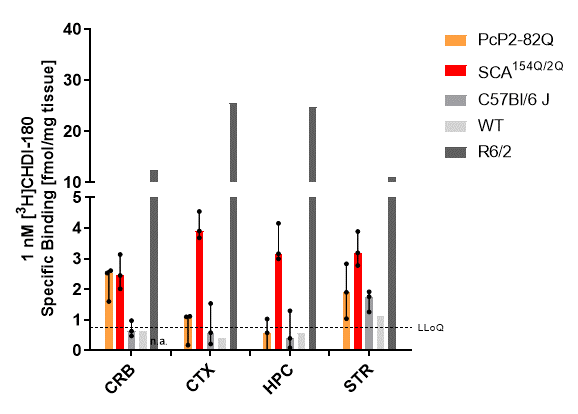


**Figure S11**: Autoradiographic studies investigating CHDI-180 ligand binding in Sca1 mouse models

[^3^H]CHDI-180 binding in brains of PcP2-82Q and SCA^154Q/2Q^ mice. Specific binding (SB) of 1 nM [^3^H]CHDI-180 in cerebellum (CRB), striatum (STR), cortex (CTX) and hippocampus (HPC) of 10-month-old PcP2-82Q (n=3) and 6 month-old SCA^154Q/2Q^ brain. One R6/2 CAG120 brain at the age of 3 month was included for comparative reasons. Data shown as median ± range.

**Table S1**: Demographic information for human HD, AD and control brains.

Abbreviations: AD = Alzheimer’s disease, ARG = autoradiography, F = female, HD = Huntington’s disease, M = male, n/a = not available, NFT = neurofibrillary tangles, NBB = Netherland Brain Bank, NYBB = New York Brain Bank, PMI = postmortem interval, TS = Tissue Solutions Ltd. All samples were from frontal cortices.

| ID | Source | Diagnosis | Gender | Age at death (yrs) | PMI (hrs) | CAG repeats | Von Sattel | Braak (NFT) | Application |
| --- | --- | --- | --- | --- | --- | --- | --- | --- | --- |
| T-343 | NYBB | Control | M | 62 | 8 | n/a | n/a | n/a | ARG |
| T-4434 | NYBB | Control | F | 79 | 5 | n/a | n/a | n/a | ARG |
| T-638 | NYBB | Control | M | 78 | 8 | n/a | n/a | n/a | ARG |
| TS_17_006/007_OM_FC | TS | Control | M | 65 | 6 | n/a | n/a | n/a | ARG |
| TS_17_002/003_VQ_FC | TS | Control | M | 55 | 4 | n/a | n/a | n/a | ARG |
| TS_17_005_QK_FC | TS | Control | M | 56 | 5.5 | n/a | n/a | n/a | ARG |
| TS_17_077_078_DZ_FC | TS | Control | F | 56 | 6.5 | n/a | n/a | n/a | ARG |
| TS_17_014_WH_FC | TS | Control | M | 57 | 6.5 | n/a | n/a | n/a | ARG |
| 2014-043 | NBB | Control | F | 60 | 8 | n/a | n/a | 0 | ARG |
| 2017-005 | NBB | Control | F | 60 | 5.5 | n/a | n/a | 0 | ARG |
| T-1960 | NYBB | HD | F | 65 | 18 | 44/21 | 3 | n/a | ARG |
| T-1991 | NYBB | HD | M | 53 | 11 | 46/17 | 3/4 | n/a | ARG |
| T-2476 | NYBB | HD | M | 60 | 23 | 43/17 | 3/4 | n/a | ARG |
| T-4501 | NYBB | juvenile HD | F | 8 | 22 | 105/22 | 4/4 | n/a | ARG |
| T-5128 | NYBB | HD | M | 39 | 6 | 19/53 | 3/4 | n/a | ARG |
| T-2019 | NYBB | HD | M | 61 | 18 | 43/19 | 3/4 | n/a | ARG |
| T-5263 | NYBB | HD | F | 55 | 30 | 45/30 | 2/4 | n/a | ARG |
| 2009-063 | NBB | HD | F | 64 | 5 | n/a | n/a | n/a | ARG |
| 1999-120 | NBB | HD | M | 79 | 6 | n/a | n/a | 1 | ARG |
| 2017-060 | NBB | HD | F | 57 | 6.5 | n/a | n/a | 0 | ARG |
| 2008-044 | NBB | HD | M | 59 | 5 | n/a | n/a | n/a | ARG |
| 1995-060 | NBB | HD | M | 57 | 7.5 | n/a | n/a | n/a | ARG |
| 2013-076 | NBB | HD | M | 71 | 4.5 | n/a | n/a | 2 | ARG |
| TS_18_020_FX_FC | TS | AD | M | 70 | 8 | n/a | n/a | 6 | ARG |
| TS_18_001_DQ_FC | TS | AD | M | 80 | 7 | n/a | n/a | 5 | ARG |
| TS 18_016_MC_FC | TS | AD | M | 71 | 4.6 | n/a | n/a | 6 | ARG |
| TS_18_001_OP_FC | TS | AD | M | 84 | 6.1 | n/a | n/a | 5 | ARG |
| TS_18_015_EQ_FC | TS | AD | M | 74 | 7.7 | n/a | n/a | 5 | ARG |

**Table S2**: Autoradiographic summary of [^3^H]CHDI-180 binding in HOM zQ175 and WT brain sections.

| Age at sacrifice | Genotype | ROI | TB  [fmol/mg ± SD] | | | NSB [fmol/mg ± SD] | | SB [fmol/mg ± SD] |  |
| --- | --- | --- | --- | --- | --- | --- | --- | --- | --- |
| 3 month | HOM Q175 | STR | 3.9 ± 0.5 | | | 0.4 ± 0.4 | | 3.5 ± 0.3 |  |
|  |  | CTX | 5.6 ± 0.3 | | | 1.3 ± 2.2 | | 4.3 ± 2.3 |  |
|  |  | HPC | 5.1 ± 0.3 | | | 0.3 ± 0.3 | | 4.8 ± 0.4 |  |
| 3 month | WT | STR | 1.8 ± 0.3 | | | 0.7 ± 0.6 | | 1.1 ± 0.4 |  |
|  |  | CTX | 1.9 ± 0.5 | | | 0.6 ± 0.5 | | 1.3 ± 1.0 |  |
|  |  | HPC | 2.0 ± 0.4 | | | 0.5 ± 0.6 | | 1.6 ± 0.6 |  |
| 6 month | HOM Q175 | STR | 16.1 ± 2.9 | | | 0.3 ± 0.2 | | 15.8 ± 2.7 |  |
|  |  | CTX | 24.6 ± 1.0 | | | 0.2 ± 0.4 | | 24.4 ± 1.4 |  |
|  |  | HPC | 25.1 ± 1.6 | | | 0.1 ± 0.0 | | 25.0 ± 1.6 |  |
| 6 month | WT | STR | 2.0 ± 0.6 | | | 0.4 ± 0.5 | | 1.6 ± 0.1 |  |
|  |  | CTX | 1.8 ± 0.1 | | | 0.5 ± 0.6 | | 1.2 ± 0.5 |  |
|  |  | HPC | 2.2 ± 0.7 | 0.6 ± 0.8 | | | | 1.5 ± 0.1 |  |
| 9 month | HOM Q175 | STR | 28.8 ± 2.7 | | 0.4 ± 0.4 | | 28.4 ± 2.4 | | |
|  |  | CTX | 49.7 ± 7.4 | | 0.9 ± 0.8 | | 48.8 ± 8.3 | | |
|  |  | HPC | 41.5 ± 2.4 | | 0.2 ± 0.2 | | 41.3 ± 2.2 | | |
| 9 month | WT | STR | 1.6 ± 0.1 | | 0.0 ± 0.0 | | 1.6 ± 0.1 | | |
|  |  | CTX | 1.3 ± 0.4 | | 0.1 ± 0.2 | | 1.2 ± 0.2 | | |
|  |  | HPC | 1.8 ± 0.5 | | 0.6 ± 0.8 | | 1.1 ± 0.5 | | |
| 12 month | HOM Q175 | STR | 41.6 ± 3.1 | | 0.4 ± 0.3 | | 41.3 ± 2.8 | | |
|  |  | CTX | 70.9 ± 11.0 | | 0.8 ± 0.7 | | 70.2 ± 10.5 | | |
|  |  | HPC* | 68.7 ± 7.7 | | 0.4 ± 0.7 | | 68.7 ± 7.7 | | |
| 12 month | WT | STR | 1.3 ± 0.0 | | 0.2 ± 0.2 | | 1.2 ± 0.2 | | |
|  |  | CTX | 1.3 ± 0.3 | | 0.1 ± 0.1 | | 1.2 ± 0.2 | | |
|  |  | HPC | 1.6 ± 0.4 | | 0.0 ± 0.1 | | 1.6 ± 0.5 | | |

Total binding (TB), non-specific binding (NSB) and specific binding (SB) of 0.5 nM [^3^H]CHDI-180 in striatum (STR), cortex (CTX) and hippocampus (HPC) from 3-, 6-, 9- and 12-month-old HOM zQ175 and age-matched WT mice. Data are depicted as Mean ± SD from a total of n=3 animals per genotype. * n=2 (1 sample excluded due to tissue quality reasons).

**Supplementary Material and Methods:**

*Expression and purification of recombinant proteins*

For generation of GST-Q46, a GST-tag was fused to a polyglutamine peptide with 46 codon-optimized repeats (GST-Q46-pGEX4T1) to maintain the protein in a soluble state. A thrombin-cleavage site incorporated between the GST tag and the polyQ46 stretch allowed subsequent cleavage of GST by thrombin protease, leading to the aggregation of polyQ46.

Bacterial expression plasmid GST-Q46-pGEX4T1 was transformed by heat shock into BL21 (DE3) cells for expression. The cells were grown in LB including carbenicillin (100 µg/mL) at 37°C for 20 hrs in a shaker (120 rpm) to an OD of 4.9 (600 nm). Pre-cultures were diluted into TB including carbenicillin (100 µg/mL) to a starting OD of ~0.1 (600 nm) and grown at 37°C to an OD of ~1.0 (600 nm). 1 mM Isopropyl 1-thio-β-d-galactopyranoside (IPTG) was added and the cells were grown for 4 hrs at 37°C. Pellets were collected by centrifugation at 3000 × g for 15 min at 4°C and snap frozen at -80°C.

For purification, the pellets were thawed at room temperature and resuspended in cold lysis buffer (20 mM Tris-HCl, pH 8.0; 150 mM NaCl including protease Inhibitor tablets (Complete, EDTA-free; Roche Diagnostics, 1 tablet in 50 mL buffer) and DNAse (Benzonase, 10 U/mL)). Bacteria were lysed using a Constant System Cell Disrupter (2 cycles at 25000 psi) and lysates were cleared by centrifugation for 45 min at 27000 × g. Proteins from lysates were purified by batch binding (1 hr at 4°C) using a Glutathione Sepharose 4 FF column (GE Healthcare) and washed with several column volumes of 20 mM Tris-HCl, pH 8.0; 150 mM NaCl. Purified proteins were eluted with 20 mM Tris-HCl, pH 8.0; 150 mM NaCl, 5 mM reduced Glutathione at a flow rate of 2 mL/min. Peak fractions were analyzed by SDS-PAGE and GST-Q46 containing fractions were pooled. Purified proteins were dialyzed over night at 4°C against 10 mM TrisHCl pH 8.0, 100mM NaCl buffer. Purified and dialysed proteins were aliquoted, snap frozen in liquid nitrogen and stored at −80°C.

For generation of MBP-HTT(1-89)Q46-His(6x), a maltose binding protein (MBP)-tag followed by a thrombin-cleavage site was fused to exon 1 (aa1-89) of the human huntingtin protein with 46 codon-optimized polyglutamine repeats (pTrilJ-MV-polyQ46Exon1_His) to maintain the protein in a soluble state.

Bacterial expression plasmid pTrilJ-MV-polyQ46Exon1_His was transformed by heat shock into BL21 (DE3) cells for expression. The cells were grown in LB including carbenicillin (100 µg/mL) at 37°C in a shaker (140 rpm) to an OD of 1.5 (600 nm). Pre-cultures were diluted into TB including carbenicillin (100 µg/mL) to a starting OD of 0.02 (600 nm) and grown at 37°C to an OD of 0.5-0.6 (600 nm). 1 mM Isopropyl 1-thio-β-d-galactopyranoside (IPTG) was added and the cells were grown at 18°C for 18 hrs. Pellets were collected by centrifugation at 3000 × g for 15 min at 4°C and frozen at -80°C.

For purification the pellets were thawed at room temperature and resuspended in cold lysis buffer (20 mM Tris-HCl; pH 8.0, 300 mM NaCl including protease Inhibitor tablets (Complete, EDTA-free; Roche Diagnostics, 1 tablet in 50 mL buffer) and DNAse (4 U/mL)). Bacteria were lysed using a microfluidizer (2 cycles at 17000 psi) and lysates were cleared by centrifugation at 38000 × g for 30 min. Proteins from lysates were purified using a nickel-nitrilotriacetic acid-agarose (Ni-NTA) column (HisTrap FF Crude, GE Healthcare) at a flow rate of 4 mL/min and washed with several column volumes of 20 mM Tris-HCl, pH 8.0; 300 mM NaCl, 10 mM imidazole. Purified proteins were eluted with 20 mM Tris-HCl, pH 8.0; 300 mM NaCl, 250 mM imidazole. Pooled eluate was run over a self-packed XK 50/20 column (GE Healthcare) with 50 mL Amylose resin (NEB) at a flow rate of 2 mL/min and washed with several column volumes of 20 mM Tris-HCl, pH 8.0, 200 mM NaCl. Purified proteins were eluted with 20 mM Tris-HCl, pH 8.0, 200 mM NaCl, 10 mM Maltose at a flow rate of 2 mL/min and dialyzed over night at 4°C against 10mM TrisHCl pH 8.0, 100mM NaCl buffer. Purified and dialysed proteins were aliquoted, snap frozen in liquid nitrogen and stored at −80°C.

*Characterization of recombinant aggregates*

*Thioflavin S (ThS) binding assay*: 100 µL from several steps during the aggregation process (see Supplementary Fig. S2 B,C) was supplemented with 10 µM of ThS (T1892 SIGMA), then, the ThS fluorescence was measured using FLUOstar Omega® plate reader with excitation/emission filters of 440 nm/480 nm^1^.

*Filter-trap assay (Immunoblot)*: The formation of mHTT Exon1-Q46 or Q46 from the different aggregate preparations (see Supplementary Fig. S2D) was also assessed using a filter-trap assay, as previously reported^2^. Briefly, 20 µL of each solution was blotted on a 0.2 µm cellulose acetate membrane filter, using a 96-well dot-blot apparatus (Bio-Dot Apparatus #1706545; Bio-Rad Laboratories) under vacuum aspiration. After three washes with TBS/1% SDS, the membrane was blocked, incubated with the MW1 primary antibody^3,4^ and then with a secondary Fluor 680-conjugated polyclonal goat anti-mouse antibody. Finally, the membrane was imaged using the Odyssey Infrared Imaging System (LI-COR).

*Electron microscopy*: For transmission electron microscopic (TEM) analysis (see Supplementary Fig. S2E), 5 µL of each solution was spotted and stained using 0.7% (w/v) uranyl formate onto a Formvar/carbon-coated 200-mesh glow-discharged copper grid as previously described^5^. Imaging was performed on a Tecnai Spirit BioTWIN electron microscope equipped with a LaB6 gun and a 4K x 4K FEI Eagle CCD camera (FEI) and operated at 80 kV.

*Immunohistochemistry (IHC) in HOM zQ175 mice*

For immunostaining of HTT aggregates in HOM zQ175 mice, frozen 20 µm-thick mouse brain sections were dried in an oven for 15 min at 40°C. After fixation with 4% paraformaldehyde (PFA) for 20 min at room temperature, sections were washed 3 x 5 min in PBS and treated with 70% formic acid for 10 min for epitope retrieval. The sections were again washed 3 x 5 min in PBS and permeabilized in PBS containing 0.2% Triton X-100 for 20 min at room temperature, followed by blocking with 10% normal goat serum for 30 min at room temperature. After blocking, the sections were incubated in PBS containing 1% normal goat serum and 0.2% Triton X-100 with primary antibody mEM48 (Millipore, MAB5374; 1:100) at 4°C overnight. Sections were washed 3 x 5 min with PBS containing 0.1% Triton X-100, followed by an incubation for 2 hrs with the secondary CF568-conjugated antibody (Sigma, SAB4600082; 1:1000) in PBS with 3% normal goat serum.

The sections were again washed 3 x 10 min with PBS containing 0.1% Triton X-100 followed by a rinse in dH_2_O, and incubated for 2 min in 70% Ethanol /1% Sudan Black B (Sigma, #199664) followed by a rinse in 70% Ethanol. After short rinses in dH_2_O and PBS, sections were counterstained with DAPI (Sigma, #D9542; 1:10000) for 2 min at room temperature. Sections were again washed 3 x 5 min in PBS, cover-slipped with mounting medium Fluoromount G and dried for 1 hr at room temperature.

Images were acquired using a Zeiss Axio Scan.Z1 slide scanner with a 40x air objective. Automated image analysis was generally performed as described^6^. Density of mHTT aggregates was quantified and plotted as a ratio of mHTT nuclear inclusions per μm^2^.

*mHTT MSD assays*

mHTT MSD assays were generally performed as described^7,8^. Antibodies used for aggregated mHTT-assay: Capture antibody MW8^4,9^, 4 µg/mL in coating buffer (15 mM Na_2_CO_3_/35 mM NaHCO_3_, pH 9.6); detection antibody SULFO-TAG (ST)-labelled 4C9^10,11^, 1 µg/mL in blocking buffer (Meso Scale Discovery). Antibodies used for soluble mHTT-assay: Capture antibody 2B7^12^, 5 µg/mL in coating buffer; detection antibody ST-labelled MW1^3,4^, 5 µg/mL in blocking buffer. Both detection antibodies were labelled with a ST according to manufacturer’s instructions.

Briefly, MSD 384-well plates (Meso Scale Discovery) were coated overnight at 4°C with 10 µL of the respective coating antibody in carbonate-bicarbonate coating buffer (15 mM Na_2_CO_3_, 35 mM NaHCO_3_, pH 9.6) per well. Plates were washed three times with 35 µL of washing buffer (0.2% Tween-20 in PBS) per well and blocked with 35 µL of blocking buffer (2% probumin, 0.2% Tween-20 in PBS) per well for 1 hr at room temperature with rotational shaking at 300 rpm.

For the mHTT aggregate-specific MSD assay, brain homogenates were diluted to a final concentration of 1 mg/mL total protein in blocking buffer. For the soluble mHTT MSD assay brain extracts were first diluted in brain lysis buffer to a concentration of 0.2 mg/mL total protein and then further diluted to 0.1 mg/mL total protein in blocking buffer.

After an additional washing step, 10 µL per sample were transferred to each well of the antibody-coated MSD plate and incubated with shaking for 1 hr at room temperature. After disposal of input liquid and three wash cycles with 35 µL of washing buffer each, 10 µL of the primary detection antibody (ST labeled) were added to each well and incubated with shaking for 1 hr at room temperature. After washing three times with washing buffer, 35 µL of reading buffer T with surfactant (Meso Scale Discovery) were added to each well.

Plates were imaged on a Sector Imager 6000 (Meso Scale Discovery) according to manufacturer’s instructions and settings recommended for 384-well plates.

**References**

1 Chiki, A. *et al.* Mutant Exon1 Huntingtin Aggregation is Regulated by T3 Phosphorylation-Induced Structural Changes and Crosstalk between T3 Phosphorylation and Acetylation at K6. *Angew Chem Int Ed Engl* **56**, 5202-5207, doi:10.1002/anie.201611750 (2017).

2 Wanker, E. E. *et al.* Membrane filter assay for detection of amyloid-like polyglutamine-containing protein aggregates. *Methods Enzymol* **309**, 375-386, doi:10.1016/s0076-6879(99)09026-6 (1999).

3 Khoshnan, A., Ko, J. & Patterson, P. H. Effects of intracellular expression of anti-huntingtin antibodies of various specificities on mutant huntingtin aggregation and toxicity. *Proc Natl Acad Sci U S A* **99**, 1002-1007, doi:10.1073/pnas.022631799 (2002).

4 Ko, J., Ou, S. & Patterson, P. H. New anti-huntingtin monoclonal antibodies: implications for huntingtin conformation and its binding proteins. *Brain Res Bull* **56**, 319-329, doi:10.1016/s0361-9230(01)00599-8 (2001).

5 Booth, D. S., Avila-Sakar, A. & Cheng, Y. Visualizing proteins and macromolecular complexes by negative stain EM: from grid preparation to image acquisition. *J Vis Exp*, doi:10.3791/3227 (2011).

6 Carty, N. *et al.* Characterization of HTT inclusion size, location, and timing in the zQ175 mouse model of Huntington's disease: an in vivo high-content imaging study. *PLoS One* **10**, e0123527, doi:10.1371/journal.pone.0123527 (2015).

7 Macdonald, D. *et al.* Quantification assays for total and polyglutamine-expanded huntingtin proteins. *PLoS One* **9**, e96854, doi:10.1371/journal.pone.0096854 (2014).

8 Reindl, W. *et al.* Meso scale discovery-based assays for the detection of aggregated huntingtin. *PLoS One* **14**, e0213521, doi:10.1371/journal.pone.0213521 (2019).

9 Legleiter, J. *et al.* Monoclonal antibodies recognize distinct conformational epitopes formed by polyglutamine in a mutant huntingtin fragment. *J Biol Chem* **284**, 21647-21658, doi:10.1074/jbc.M109.016923 (2009).

10 Baldo, B. *et al.* TR-FRET-based duplex immunoassay reveals an inverse correlation of soluble and aggregated mutant huntingtin in huntington's disease. *Chem Biol* **19**, 264-275, doi:10.1016/j.chembiol.2011.12.020 (2012).

11 Landles, C. *et al.* Proteolysis of mutant huntingtin produces an exon 1 fragment that accumulates as an aggregated protein in neuronal nuclei in Huntington disease. *J Biol Chem* **285**, 8808-8823, doi:10.1074/jbc.M109.075028 (2010).

12 Weiss, A. *et al.* Single-step detection of mutant huntingtin in animal and human tissues: a bioassay for Huntington's disease. *Anal Biochem* **395**, 8-15, doi:10.1016/j.ab.2009.08.001 (2009).
